# Identifying environmental versus phylogenetic correlates of behavioural ecology in gibbons: implications for conservation management of the world’s rarest ape

**DOI:** 10.1186/s12862-015-0430-1

**Published:** 2015-08-25

**Authors:** Jessica V. Bryant, Valérie A. Olson, Helen J. Chatterjee, Samuel T. Turvey

**Affiliations:** Institute of Zoology, Zoological Society of London, Regent’s Park, London, NW1 4RY UK; Department of Genetics, Evolution and Environment, University College London, Gower Street, London, WC1E 6BT UK; Care Quality Commission, 103-105 Bunhill Row, London, EC1Y 8TG UK

**Keywords:** Comparative analysis, Group size, Hainan gibbon, Home range, Hylobatidae, Lambda, Mating system, *Nomascus hainanus*, Phylogenetic signal

## Abstract

**Background:**

For conservation of highly threatened species to be effective, it is crucial to differentiate natural population parameters from atypical behavioural, ecological and demographic characteristics associated with human disturbance and habitat degradation, which can constrain population growth and recovery. Unfortunately, these parameters can be very hard to determine for species of extreme rarity. The Hainan gibbon (*Nomascus hainanus*), the world’s rarest ape, consists of a single population of c.25 individuals, but intensive management is constrained by a limited understanding of the species’ expected population characteristics and environmental requirements. In order to generate a more robust evidence-base for Hainan gibbon conservation, we employed a comparative approach to identify intrinsic and extrinsic drivers of variation in key ecological and behavioural traits (home range size, social group size, mating system) across the Hylobatidae while controlling for phylogenetic non-independence.

**Results:**

All three studied traits show strong phylogenetic signals across the Hylobatidae. Although the Hainan gibbon and some closely related species have large reported group sizes, no observed gibbon group size is significantly different from the values expected on the basis of phylogenetic relationship alone. However, the Hainan gibbon and two other *Nomascus* species (*N. concolor*, *N. nasutus*) show home range values that are higher than expected relative to all other gibbon species. Predictive models incorporating intraspecific trait variation but controlling for covariance between population samples due to phylogenetic relatedness reveal additional environmental and biological determinants of variation in gibbon ranging requirements and social structure, but not those immediately associated with recent habitat degradation.

**Conclusions:**

Our study represents the first systematic assessment of behavioural and ecological trait patterns across the Hylobatidae using recent approaches in comparative analysis. By formally contextualising the Hainan gibbon’s observed behavioural and ecological characteristics within family-wide variation in gibbons, we are able to determine natural population parameters expected for this Critically Endangered species, as well as wider correlates of variation for key population characteristics across the Hylobatidae. This approach reveals key insights with a direct impact on future Hainan gibbon conservation planning, and demonstrates the usefulness of the comparative approach for informing management of species of conservation concern.

**Electronic supplementary material:**

The online version of this article (doi:10.1186/s12862-015-0430-1) contains supplementary material, which is available to authorized users.

## Background

In order to maximise the effectiveness of conservation interventions for threatened species, it is necessary to understand not only the extrinsic factors responsible for past or current declines, but also the intrinsic characteristics that such species would be expected to display under optimal environmental conditions. However, remnant populations of threatened species may exhibit atypical behavioural, ecological and demographic characteristics that can restrict population growth and recovery, for example due to geographical restriction to environmental refugia containing sub-optimal habitat [1, 2], or disruption of mating systems by Allee effects (fitness impacts associated with population size) beyond low population density thresholds [3]. Indeed, a fundamental concept in behavioural ecology is that animals will respond to environmental changes first through modifications in behaviour, with adaptations in life history, physiology and morphology occurring over longer periods [4], such that flexibility in key behavioural traits may be expected in response to recent habitat changes due to human disturbance and degradation. It is of particular importance to determine natural population parameters for highly threatened species that have been reduced to only a handful of surviving individuals, as such species of extreme rarity will likely require urgent and intensive conservation management [5]. Unfortunately, these parameters may be particularly hard to determine for such species; for example, direct assessment of variation in population-level responses to different environmental conditions is impossible if a species of concern is now restricted to a single site. Successful evidence-based conservation of species of extreme rarity may therefore necessitate using alternative investigative approaches to determine their expected natural behavioural, ecological and demographic characteristics and environmental requirements.

The Hainan gibbon (*Nomascus hainanus*) is the world’s rarest ape, rarest primate, and possibly rarest mammal species, consisting of a single population of c.25 individuals constrained to Bawangling National Nature Reserve on Hainan Island, China. This population persists in a small (c.15 km^2^) area of fragmented, relatively high-elevation forest that may represent suboptimal gibbon habitat [6]. Conservation efforts for the species have focused predominantly on mitigating external factors responsible for past population decline, together with ongoing monitoring [6–8]. More intensive, active management has been largely constrained by a deficit of robust data and general lack of clarity regarding even the species’ basic ecology. Crucially, it remains unclear which ecological and behavioural characteristics observed in the tiny remnant Hainan gibbon population may be “natural” for the species even before human impact, and which may be artefacts of the population’s currently compromised situation. This lack of data represents a major barrier to effective conservation planning, as it is difficult to assess which factors might be managed and which aspects of the species’ biology are unlikely to change despite intensive management.

Gibbons (Hylobatidae) are generally considered to show relatively consistent patterns of diet, territory and home range size, group composition and mating strategy, despite occurring across different forest environments [9]. They typically occur in small monogamous social groups, consisting of a nuclear family containing an adult male, adult female and 1–3 offspring, which maintain relatively small home ranges of c.0.40 km^2^ [9, 10]. The Hainan gibbon appears remarkable in terms of these characteristics, with large polygynous groups (typically >6 individuals, with observations of up to 12 individuals in one group) maintaining much larger home ranges (estimates between c.1.5–10 km^2^) [11–13]. The drivers behind these apparently anomalous ranging and social habits are not clear. These features may constitute typical characteristics of Hainan gibbon biology [11, 14]. Alternately, they may constitute the current population’s response to extrinsic pressures, with reduced habitat availability and suboptimal habitat quality potentially driving large home range requirements, which prevents establishment of new social groups and forces individuals to remain within their natal groups [6, 7, 12]. Limited mating opportunities generated by the greatly restricted current population size may explain the observed polygynous mating system as an abnormal behaviour [6, 15], and a lack of neighbouring groups may permit expansion of existing group home ranges [7].

Understanding the intrinsic versus extrinsic drivers of these behaviours to inform conservation efforts for the Hainan gibbon requires wider consideration of ecological and behavioural patterns observed under different environmental conditions across other gibbon species. The conventional description of gibbon ranging and social organisation is largely based on historical studies of *Hylobates* [16]. However, studies of wild populations of increasing numbers of species have revealed that while these general habits may still be predominant, there is greater variation in gibbon ecology and behaviour than originally supposed. Occurrences of single social groups containing >2 adults comprise at least 10 % of all groups studied [17], and within-family variability in group size and ranging behaviour is also documented [18], with other highly threatened gibbons (*Nomascus* and *Hoolock* species) in particular showing considerable variation in these characteristics [19–21]. These observations could therefore support either the disturbance hypothesis of anthropogenic pressure and habitat alteration regulating gibbon behaviour, with flexibility in gibbon behavioural ecology in response to extrinsic drivers [18], or alternately the existence of intrinsic phylogenetically-driven patterns of ecological and behavioural variation within the Hylobatidae.

Despite the importance of clarifying the drivers of Hainan gibbon ecology and behaviour for best-practice conservation planning, there has been no formal attempt to contextualise the species’ home range and social organisation beyond simple descriptive comparisons with other gibbons [6]. The comparative approach, whereby correlations between variables are investigated statistically while controlling for phylogenetic non-independence, has been widely employed to inform conservation efforts. Macroecological comparative analyses have been used to identify broad-scale predictors of extinction risk and population decline [22–24], and which species are more likely to benefit from intensive intervention actions such as captive breeding [25] and translocation [26]. Comparative analyses at the family level can identify intrinsic and extrinsic drivers of variation in ecological and behavioural traits seen within individual groups of interest [27–29], thus representing a valuable tool to inform management of species of conservation concern.

In order to generate a more robust evidence-base for Hainan gibbon conservation, we employed a comparative approach to identify drivers of home range size, social group size and mating system across different gibbon species. This analysis represents the first systematic attempt to determine the contribution of intrinsic versus extrinsic factors, and the evidence for phylogenetic versus non-phylogenetic control, to variation in key behavioural, ecological, and demographic traits across the Hylobatidae. Our results are of direct relevance to Hainan gibbon conservation management, and have wider implications for understanding gibbon ecology.

## Methods

### Data collection

A comparative dataset on home range (HR), group size (GS) and mating system (MS) was compiled for the 19 currently recognised gibbon species [9], including observations for as many gibbon populations (separate study sites) as possible to capture intraspecific as well as interspecific variation, and permit detection of extrinsic, site-level influence as well as phylogenetic influence upon expression of response traits. Hainan gibbon GS values were derived from the most recent available data for the species [13]; to account for variation in available estimates of Hainan gibbon HR, separate analyses were conducted using two differing recent estimates of HR for this species: a high estimate of 7.67 km^2^ [12] and a much lower estimate of 1.48 km^2^ [13]. Other data were obtained from published and grey literature and by surveying gibbon researchers with knowledge/experience across a range of field sites. This combined approach captured comparative data for 39 populations across the 19 species (Additional file 1); data from >1 population were obtained for 58 % of species, with further data collection limited in some cases by the existence of only a single extant or studied population per species.

Site-specific data for 11 potential predictors were collected for use in predictive modelling (Table 1). To avoid over-parameterisation, only data on key intrinsic and extrinsic variables hypothesised *a priori* to influence our response traits were incorporated [30, 31]. Fruit availability and load may be an important predictor of HR size, with GS and group density increasing in locations with higher fruit tree densities [9, 32, 33]. Site-specific information on gibbon food tree densities was generally unavailable; we used data on a series of extrinsic variables (latitude, longitude, altitude, annual mean temperature, annual precipitation) that capture site climatic conditions and potential productivity and are likely to determine food tree density for gibbons, as has been demonstrated in species-specific studies at smaller scales [34, 35] and for Asian tropical forests more generally [36]. An additional, more direct measure of productivity, the normalized difference vegetation index (NDVI; a satellite-based vegetation index derived from the red:near-infrared reflectance ratio of light reflected by vegetation and captured by satellite sensor, which correlates strongly with above-ground net primary productivity), was also included as a surrogate for vegetation structure and therefore annual productivity and biomass [37, 38]. Seasonal variation in resources has been hypothesised to determine gibbon HR and number of defendable females [39], with gibbons possibly requiring larger areas to obtain resources in more seasonal environments, relaxing constraints on GS [9]; NDVI correlates with seasonal average energy availability [40, 41], and seasonality in precipitation was also included to further capture inter-site variation in seasonality. Low group density has been proposed as a possible explanation for the apparently high Hainan gibbon HR and GS as a result of greater space available for the small population [6, 7, 12], although delayed dispersal from groups and thus potentially larger GS may occur in habitats saturated with gibbon groups [42]. Gibbon group density and reserve area (in lieu of generally unavailable data on gibbon-suitable habitat availability) were therefore included as measures of potential intraspecific site-specific competition for space and resources. Global Human Footprint (GHF) data, comprising a composite index of relative human influence derived from human population density, land use and infrastructure [43], were used as a standardised proxy for anthropogenic disturbance. Adult body mass was included as a proxy for various life-history traits that may interact with and influence numerous ecological and behavioural characteristics in mammals (e.g. reproductive rate, gestation period, weaning length, interbirth interval [22, 23]); no site-level body mass data were available, so species mean data (where available), or medians calculated from a range of reported body masses, were replicated across all populations of that species.

**Table 1:** Potential predictor variables of home range, group size and mating system in gibbons: potential intrinsic and extrinsic predictor variables (fixed effects) hypothesised to influence response traits and tested in predictive models, with data scale and source(s)

Potential predictor variable	Scale	Source(s)
Adult body mass (kg)	Species mean	[9, 50, 51, 86, 87]
Group density (mean number of groups/km^2^)	Mean at site	Details in Additional file 1
Latitude (decimal degrees)	Exact value for site	Details in Additional file 1
Longitude (decimal degrees)	Exact value for site	Details in Additional file 1
Altitude (metres asl)	Mean for site across years, 1 km resolution	[88], extracted in ArcMap V.10.0 (ESRI 1999–2010); site location used to derive standardised mean values per site
Annual mean temperature (°C)	Mean for site across years, 1 km resolution	
Annual precipitation (mm)	Mean for site across years, 1 km resolution	
Precipitation seasonality (coefficient of variation)	Mean for site across years, 1 km resolution	
Normalized difference vegetation index (NDVI; ratio)	Mean for site across years, 8 km resolution	[89]
Global Human Footprint (GHF; %)	Mean for site across years, 1 km resolution	[43]
Reserve area (km^2^)	Value for reserve	Details in Additional file 1

### Phylogenetic tree

To incorporate phylogenetic relatedness between gibbon species into our analysis, we manually reconstructed a phylogenetic tree for the Hylobatidae within TreeEdit V.1.0a10 [44]. We recognise that a recent study [45, 46], which incorporated only five species (two for *Hylobates*, and one each for the other three gibbon genera), has suggested that it is difficult to resolve the pattern of phylogenetic branching within Hylobatidae or identify any strongly-supported single tree topology due to rapid initial radiation of all four gibbon genera. However, the only recent phylogenetic study to incorporate all then-recognised gibbon taxa, and the only published gibbon phylogeny to include the Hainan gibbon [47], was able to identify a single best-supported tree, although support for some branches was low. To account for this phylogenetic uncertainty, we investigated the impact of alternative tree topologies upon the output of our principal analysis, and showed that output signal strength remained consistent across a range of available phylogenetic trees representing different possible relationships among gibbons (see Additional file 2). Therefore, we retained the most complete gibbon tree topology [47] as the basis for our comparative analyses. Within this study, the small dataset (*n* = 19 species) prevented the use of more advanced methods to derive alternative tree topologies (e.g. via phylogenetic estimation resampling).

**Fig. 1:**
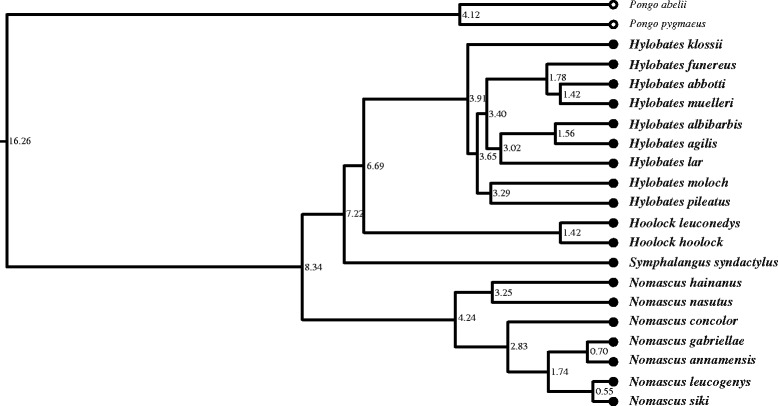
Reconstructed hylobatid phylogenetic tree (with outgroup): phylogenetic tree of the Hylobatidae plus *Pongo* outgroup (indicated by hollow circles) used for tests of phylogenetic signal and predictive modelling, with mean divergence times (in mya) indicated at nodes; after [47] and Christian Roos (pers. comm., September 2013)

The phylogeny of ref. [47] was modified as follows (see Fig. 1): subspecies were excluded; *Hylobates abbotti* and *H. funereus* were recognised as separate species rather than subspecies of *H. muelleri* [9]; and a further recently described species, *Nomascus annamensis* [48], was incorporated using unpublished genetic divergence data which indicate this species split from *N. gabriellae* c.0.7 million years ago (Christian Roos pers. comm., September 2013). The lineage containing the two orangutan species (*Pongo abelii*, *P. pygmaeus*), which constitutes the next oldest divergence within Hominoidea, was included as an outgroup when testing for phylogenetic signal; comparative data on response variables were collected from published sources for both orangutan species [49–53]. Orangutans represent an ecologically relevant outgroup for this study as they occupy similar and sometimes sympatric habitats to gibbons in south-east Asia, and exhibit several key biological similarities including suspensory locomotory behaviours and similar diets [53].

### Data analysis

Continuous variables that varied by an order of magnitude were log-transformed prior to analysis to normalise distributions and equalise error variance. GHF values were reduced to proportions and Arcsine transformed. Absolute latitude values were employed. The categorical response variable MS was binary-coded to focus on the presence or absence of polygyny within gibbons (polygyny = 1; monogamy = 0; for *Pongo* spp., solitary = 0), and due to the inherent limitations of available tests of phylogenetic signal for categorical variables which dictate use of binary categories [54]. Data transformations and all analyses were carried out in R V.3.0.1 [55].

To test for phylogenetic signal in our continuous response variables (HR and GS), we employed species average values (across-population observations), and estimated Pagel’s lambda, λ [56], under both maximum likelihood (ML) and log likelihoods at λ = 0 (phylogenetic independence) and λ = 1 (Brownian phylogenetic dependence) using phylogenetic generalized linear models (PGLMs [57]; revised version of code PGLM V.3.4 provided by R. Freckleton, October 2013). As a control, we also tested for phylogenetic signal in body mass; this trait has a significant phylogenetic signal across several bird and mammal groups [57], including primates [58], and therefore could be expected to show a similar signal in gibbons. Binary categorical variables cannot be tested using λ, so for the binary trait MS, we tested for phylogenetic signal using *D* [54] implemented within the “caper” package [59]. To assess *D*, we employed the predominantly observed MS for each species, which proved to be uniform (100 % of cases) across all populations surveyed for each species.

Prior to running predictive models, correlations between predictor variables were assessed to test for collinearity using correlation matrices and simple linear models generated for each pairwise combination of predictors. All pairwise comparisons yielded absolute correlation values and variance explained by linear models (adjusted R^2^) of <0.5, indicating no issues of collinearity, so all predictors were retained. Predictive regression models were constructed using the linear mixed-effects kinship model fit by maximum likelihood (lmekin) within the “coxme” package [60].

Following a common approach in wide-scale comparative analyses [22, 24, 61], we first conducted single predictor lmekin regressions of our response variables against each predictor variable to examine the significance of each predictor separately. As a population’s HR, GS and MS may be interlinked, with some traits potentially determining others, the response variables not being tested within a given predictive model were also used as predictors for each response in turn; in total, 13 single predictor models were therefore run per response. Predictors that were significant at α < 0.05 were then incorporated into a global model for each response variable. This approach aimed to reduce the number of predictors within each global model to avoid issues of overparameterization or overfitting of data and bias in regression coefficients; a rule of ≥5 observations to one predictor was adopted [62], which for *n =* 39 populations meant using 4–7 predictors. This precluded the incorporation of any predictor interaction terms, meaning that only main effects were fitted; given our small sample size and the absence of collinearity in our predictors, we consider this approach to be robust.

We employed an IT multimodel inference approach to model selection [30]; a set of candidate models were generated representing all unique combinations of predictors in the global model, and were ranked using Akaike’s Information Criterion (AIC) [63]. For each model, the relative log-likelihood, AICc, ΔAICc, and Akaike weights (*w*_*i*_) were calculated using the “MuMIn” package [64]. We used a model-averaging approach to check the validity of the top-ranking model in each case, only including models with ΔAICc < 7 [65]; this corresponded to a cumulative *w*_*i*_ > 0.95 in all cases, thereby constituting a 95 % confidence set [30]. The relative importance (RI) of each parameter after model-averaging was calculated by summing *w*_*i*_ across all models in which the parameter was present.

## Results

### Phylogenetic signal

All three traits of interest (HR, GS and MS) had a significant phylogenetic signal. A strong phylogenetic signal was also detected for the control variable, body mass, indicating that this analytical approach is appropriate and the results for all traits tested are valid. Values of λ for HR, GS and body mass were all close to one under ML (Table 2); each ML-λ value for these traits was significantly different from zero but not significantly different from one, meaning that λ for each trait did not differ from expected Brownian phylogenetic structure in each case. These strong, statistically significant patterns of phylogenetic signal therefore indicate that more closely related gibbon species resemble each other in terms of body mass, HR and GS more than expected by chance. These signals were consistent across additional tests employing alternative phylogenetic trees (see Additional file 2). In order to assess the potential significance of other underlying intrinsic or extrinsic drivers of these traits, it was therefore necessary to control for phylogenetic relationships within our comparative dataset, as per the lmekin predictive models.

**Table 2:** Trait phylogenetic signals: results of tests for phylogenetic signal in two continuous traits of interest (home range and group size) and one control variable (body mass) using Pagel’s λ under maximum likelihood (ML-λ) and tests against models of no signal (0) or complete phylogenetic dependence (1)

Variable	ML-λ	Test	*χ* ^2^	*P*-value
Body mass	0.9999	λ = 0	45.38	<0.0001
		λ = 1	−0.01	1.0
Home range	0.9999	λ = 0	30.32	<0.0001
		λ = 1	−0.004	1.0
Group size	0.9731	λ = 0	20.61	<0.0001
		λ = 1	0.58	0.45

To ascertain if any gibbon species display HR, GS, or body mass values significantly different to those expected from phylogenetic signal alone, the PGLM derived for each trait was used to determine expected trait values for each species based upon values observed across all other species, with expected values compared to observed values using Student’s t-tests. The resultant *P*-values (after Bonferroni correction, adjusted *P*-value = 0.0024) revealed that no body mass or GS values for any species were significantly different to those expected according to the phylogenetic signal for each trait, despite the larger group sizes reported for the Hainan gibbon and some other *Nomascus* species. However, relative to all other gibbon species, HR values are higher than expected for the Hainan gibbon (*P* = 0.001) and two other *Nomascus* species (black crested gibbon *N. concolor*, *P* = 0.001; Cao Vit gibbon *N. nasutus, P* = 0.002), even under the strong phylogenetic signal observed for this trait. These results remained the same irrespective of whether a high or low estimate for Hainan gibbon HR was used; as even the less extreme HR estimate is significantly higher than expected, we therefore present only the outputs of predictive models employing this smaller estimate. Significant HR values were also detected for both *Pongo* species (*P* < 0.0001 for both species), but this is not surprising as these species are known to exhibit very large HRs (mean *P. abelii* HR = 19 km^2^; mean *P. pygmaeus* HR = 16.75 km^2^) and our study was focused primarily on variation across the Hylobatidae.

A strong phylogenetic signal was also apparent in MS, with an estimated *D* = −1.386 indicating substantial phylogenetic clumping in the binary representation of this trait, and therefore that MS is highly phylogenetically conserved within the Hylobatidae. The associated probability of observing this *D* value was not significantly different to that simulated under Brownian phylogenetic structure (*P* = 0.945), but was significantly different to that estimated under no phylogenetic structure (*P* < 0.01), indicating that the observed phylogenetic signal is statistically significant. As *D* is calculated as a contrast rather than a linear model, it was not possible to predict expected species MS values under phylogenetic signal in this trait or compare observed versus expected values for individual species; it was therefore not possible to determine if the observed MS for any gibbon species differs from what might be expected under the observed phylogenetic signal.

### Predictive models

Five predictors (as derived from significant terms detected in the single lmekin regression models) were incorporated into each global model for HR and GS, although not all terms remained significant when incorporated into multiple regression models (Additional file 3). From the HR and GS global models, a set of 31 candidate models (including global model and single predictor models) were generated and ranked by AICc. In addition to the best-approximating model with lowest AICc, 19 models with ΔAICc < 7 were identified for HR (corresponding to a cumulative *w*_*i*_ = 0.985; Additional file 4), and 10 models with ΔAICc < 7 were identified for GS (corresponding to a cumulative *w*_*i*_ = 0.957; Additional file 4).

When covariance between population samples due to phylogenetic relatedness was taken into account, variation in HR in the best-approximating model was explained by GS, MS, and group density; larger HR was predicted by larger GS, polygynous MS, and lower group density (Table 3a). Total variance explained by random (relatedness) effects was 0.00024, with 99.6 % of variance due to phylogenetic effects and 0.43 % contributed by inter-population variation within a species. Model-averaged coefficient estimates derived from the 98.5 % model confidence set agreed with the best-approximating model, with the same three variables detected as significant predictors and each having RI values of 0.70–0.84 (Table 4a). The top-ranking GS model indicated that larger GS was explained by polygynous MS and lower annual precipitation (Table 3b); annual mean temperature, although included in the best-fitting model, was not a significant predictor of GS. Total variance explained by random effects in the final GS model was very low (3.48 × 10^−8^), with 8.5 % contributed by phylogeny and 91.5 % contributed by inter-population (within-species) variation. Model-averaged coefficient estimates derived from the 95.7 % model confidence set again agreed with the best-approximating model in terms of coefficient relative magnitude and significance, with MS and annual precipitation remaining the only significant terms and each having high RI values (1.00 and 0.87 respectively; Table 4b).

**Table 3:** Best-approximating home range and group size predictive models: fixed effects parameter estimates from best-approximating linear mixed-effects kinship models incorporating phylogenetic and within-species variance-covariance fit by maximum likelihood for: a) HR (residual error = 0.113) and b) GS (residual error: 0.045). Model fitting incorporates both fixed and random effects in parameter estimates

Coefficient	Estimate	SE	z-value	*P*-value
**a) HR**				
(Intercept)	1.03	0.22	4.66	**<0.0001**
Group density	−0.14	0.04	−3.19	**0.001**
Mating system (1 = polygyny)	0.30	0.11	2.58	**0.010**
Group size	0.99	0.40	2.49	**0.013**
**b) GS**				
(Intercept)	0.98	0.18	5.55	**<0.0001**
Mating system (1 = polygyny)	0.22	0.03	6.85	**<0.0001**
Annual precipitation	−0.16	0.06	−2.96	**0.003**
Annual mean temperature	0.005	0.003	1.59	0.110

*P*-values <0.05 are shown in bold

**Table 4:** Model-averaged home range and group size predictive models: model-averaged fixed effects parameter estimates for: a) HR (from *n* = 19 model set with ΔAICc < 7 and cumulative *w*_*i*_ >0.95), and b) GS (from *n* = 10 model set with ΔAICc < 7 and cumulative *w*_*i*_ >0.95), with relative importance (RI) of each parameter

Coefficient	Averaged estimate (β)	SE	z-value	*P*-value	RI
**a) HR**					
(Intercept)	1.35	0.58	2.33	**0.020**	NA
Group density	−0.13	0.05	2.58	**0.010**	0.70
Group size	1.22	0.50	2.44	**0.015**	0.84
Mating system (1 = polygyny)	0.33	0.15	2.22	**0.026**	0.83
Annual mean temperature	−0.02	0.01	1.51	0.132	0.43
Annual precipitation	−0.19	0.17	1.08	0.282	0.26
**b) GS**					
(Intercept)	0.94	0.23	4.03	**0.0001**	NA
Mating system (1 = polygyny)	0.21	0.03	6.29	**<0.0001**	1.00
Aannual precipitation	−0.15	0.06	2.63	**0.009**	0.87
Annual mean temperature	0.005	0.003	1.43	0.153	0.39
Latitude	−0.001	0.001	0.48	0.634	0.22

*P*-values <0.05 are shown in bold

**Fig. 2:**
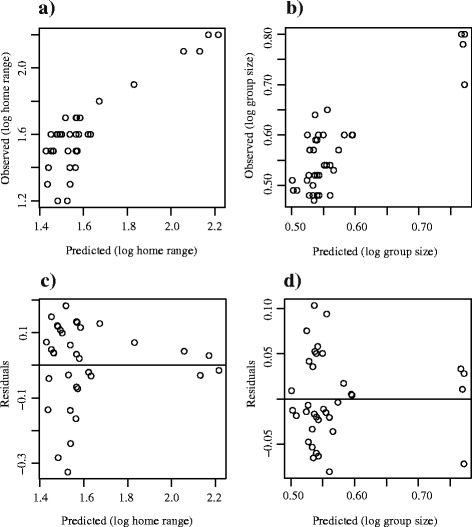
Assessment of model fit for best-approximating home range and group size predictive models: scatterplots of model fit: (**a**) observed HR values (log-transformed) versus values predicted under best-approximating linear mixed-effects kinship model for HR (log values); (**b**) observed GS values (log-transformed) versus values predicted under best-approximating linear mixed-effects kinship model for GS (log values); (**c**) HR values predicted under best-approximating linear mixed-effects kinship model for HR (log values) versus model residuals; (**d**) GS values predicted under best-approximating linear mixed-effects kinship model for GS (log values) versus model residuals

Assessment of model fit supports the validity of the best-approximating HR and GS models. Observed HR and GS values display linear trends when compared to values predicted by the top-ranking models (Fig. 2a–b), indicating that specification of main effects only did not result in poor fit due to omission of any major interaction terms. Plots of residuals versus predicted values from each model further confirms adequacy of both models, with points scattering around zero and no obvious linearity or curvature (Fig. 2c–d).

MS was not explained by any potential intrinsic or extrinsic predictor variables, whether tested in single predictor regression models or together in an exploratory global model combining all 13 potential predictors (Table 5). The variance explained by phylogeny for the random effects was infinite, indicating a strong effect of between-species phylogenetic relationships relative to within-species variation. As no significant fixed effects predictors were detected, no further analysis of this response variable was possible within the scope of this analysis.

**Table 5:** Global mating system predictive model: exploratory global multiple regression model for MS; linear mixed-effects kinship model incorporating phylogenetic and within-species variance-covariance fit by maximum likelihood (residual error: 0.129), with all possible putative fixed effect predictors

Coefficient	Estimate	SE	z-value	*P*-value
(Intercept)	0.34	0.16	2.07	**0.039**
Group size	1.36	0.50	1.86	0.063
Home range	0.50	0.23	1.82	0.069
NDVI	−0.59	0.30	−1.79	0.073
Altitude	0.16	0.10	1.72	0.085
Reserve area	−0.06	0.03	−1.66	0.097
Species adult body mass	0.45	0.33	1.38	0.170
GHF	−0.32	0.31	−1.03	0.310
Annual precipitation	−0.18	0.24	−0.73	0.460
Precipitation seasonality	−0.27	0.37	−0.74	0.460
Latitude	0.00	0.01	0.37	0.710
Longitude	0.00	0.01	0.28	0.780
Annual mean temperature	0.01	0.03	0.24	0.810
Group density	0.02	0.08	0.22	0.830

*P*-values <0.05 are in bold

### Drivers of family-wide trait patterns

Two intrinsic factors explained variability in home range across gibbon populations: mating system and group size, which were also linked. Despite the foraging advantages that gibbons gain from their energy-efficient brachiation, this positive association of home range with group size is consistent with patterns seen more widely across other primates, where larger groups will deplete food sources in discrete patches more rapidly so must range further to satisfy these greater energy requirements [66–68]. Primate species adopting polygynous mating systems will form larger groups, and so our discovery that polygynous populations are also linked to larger home ranges is likely to represent an associated effect. Accounting for mating system and group size, gibbon populations at sites with lower group densities, and therefore fewer neighbouring groups, were more likely to exhibit larger home ranges. This provides indirect evidence for a ‘disturbance’ effect regulating gibbon social organization, with lower group densities more likely to occur in threatened species due to their typically small population sizes. Within-species variation in gibbon density has also been shown to correlate with site-level vegetation parameters such as food tree availability, tree height/density, and/or canopy cover [33, 35, 69]. Site carrying capacity may therefore regulate gibbon home range size through the effects of site-level resource availability on group density, with home range size interacting with both intrinsic and extrinsic factors across the Hylobatidae.

Gibbon group size was linked to climatic as well as social drivers, with larger group sizes associated with low mean annual rainfall in addition to polygynous mating system. Drier areas predicting larger groups initially seems counterintuitive, as it is generally thought that habitats with higher levels of annual rainfall have higher productivity than drier habitats [70]. However, tropical forest productivity declines when annual rainfall exceeds 2500 mm, likely due to nutrient-leaching limiting plant growth [71]. Therefore, increased precipitation within moist tropical habitats inhabited by gibbons may negatively impact growth of gibbon food trees. Indeed, tree density and primate biomass have been shown to decline with increasing rainfall levels in southeast Asian forests [72], and frugivorous primate biomass declines with greater annual rainfall in Asia [73]. Across our sampled gibbon populations (annual precipitation range: c.1100–4000 mm), drier sites may therefore be relatively richer in available resources. Increased resource availability could permit larger group sizes by reducing intra-group feeding competition, a major cost of group living among primates [66, 74].

Beyond a strong phylogenetic signal, no explanatory variables were statistically associated with mating system. It seems unlikely that this is solely the result of limitations associated with our analytical approach; while the predictive power of a binary response model may be low, phylogenetic regression constitutes a robust analytical approach for binary response variables [75]. Our results may therefore indicate that recent extrinsic factors have limited importance for driving observed variation in gibbon mating system, with present patterns instead resulting from longer-term evolutionary processes. Monogamy is estimated to have evolved in gibbons c.19 mya [76], with monogamous mating systems in primates evolving repeatedly from polygynous systems with a zero reverse rate detected in this study. Polygyny in gibbons may therefore be unlikely to represent a recent behavioural response to compromised habitat conditions. Although several different mechanisms have been proposed to explain the evolution of mating systems in gibbons, causal links have not been demonstrated convincingly between any suggested external drivers and specific mating systems [77]. Future analyses involving approaches such as evolutionary trait mapping and ancestral character reconstruction (which were beyond the scope of this study due to taxon sampling constraints which can reduce the accuracy of trait inference [78], and limited fossil material for extinct gibbon taxa [79] required to inform trait estimation models [80]) may be helpful in clarifying evolutionary drivers and apparent flexibility in mating system across the Hylobatidae.

Both Pagel’s λ and *D* can have reduced power when dealing with small tree sizes, and high or low trait prevalence (proportions of one character state) may also reduce detection of phylogenetic signal in binary traits tested via *D*. However, false signals are unlikely under either analysis, with λ relatively robust to both tree size and uncertainty when signal strength is strong [54, 57, 81], and both tests provided strong signals (phylogenetic signals were all close to λ = 1, and *D* was high), giving us confidence in our results. Although we found evidence for both intrinsic and extrinsic drivers of gibbon ranging requirements and social group size, there was only limited support for any association with site disturbance or quality. Home range variation was explained by local group density, indirectly indicating a possible effect of carrying capacity and thus response to habitat conditions. Group size was associated with a climatic variable (annual precipitation) that may become an index of human-caused environmental change in the near future under projected climate change scenarios, but is not currently a correlate of human-caused habitat disturbance. Similarly, we found no direct support for the disturbance-hypothesis explanation for mating system. None of our selected behavioural and ecological traits were statistically linked to the proxies specifically employed to represent site habitat productivity/quality (NDVI) or disturbance (GHF). This lack of association could reflect limitations of such metrics, which may be insufficiently sensitive for capturing heterogeneity across high-biomass areas such as tropical forests at fine spatial scales [82] and will be unable to detect other key human impacts to gibbons such as localised hunting. However, while it is not possible to completely rule out a disturbance-associated effect to explain observed variations in behaviour and ecology between gibbon populations, the evidence for such an explanation is limited in contrast to the significant effect detected for other drivers.

## Discussion

**Fig. 3:**
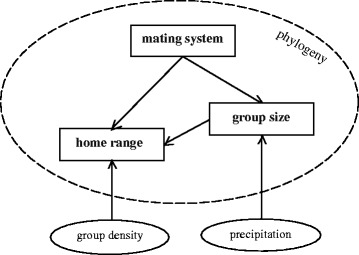
Detected drivers of home range, group size and mating system in gibbons: relationship between response variables investigated showing inter-connectivity of HR, GS and MS as both drivers and responses, along with two site-level extrinsic drivers

Our study represents the first targeted assessment of behavioural and ecological trait patterns across the Hylobatidae using recent approaches in comparative analysis. Although a small number of studies have demonstrated phylogenetic signals for selected traits in primates, including mating system [76], group size and home range [58], we present important new evidence that these traits are all strongly phylogenetically conserved within the Hylobatidae. This phylogenetic trait conservatism may be an effect associated with rapid initial evolution recently proposed for the main hylobatid lineages [45, 46]. Notwithstanding the challenges in resolving hylobatid tree topology identified in these recent studies, by accounting for this phylogenetic signal and further considering the potential effects of a range of different intrinsic and extrinsic factors on gibbon behavioural ecology both between and within species, our predictive analyses have also revealed additional environmental and biological determinants of variation in gibbon ranging requirements and social structure. It is apparent that gibbon mating system, group size and home range are inherently linked, with these factors being important, inter-correlated predictors of each other, and with further variation driven by a limited number of site-level factors (Fig. 3). A combination of social dynamics and external factors therefore seem to determine variation in these key aspects of behavioural ecology within the Hylobatidae.

In the context of the existing paradigm of gibbon ecology and behaviour, the Hainan gibbon has been regarded as displaying unusual patterns of home range and social organisation, which have often been interpreted as artefacts of anthropogenic disturbance (driven either by reduced habitat availability and/or quality, or by small population size limiting mate availability and social group formation and altering group structure [6, 7, 12, 15]). Our comparative analysis constitutes the first attempt to formally contextualise the Hainan gibbon’s observed behavioural and ecological characteristics within family-wide variation observed across gibbons in mating system, group size and home range, and our results provide important new insights for understanding the natural population parameters that would be expected for this species.

Irrespective of whether we use high or low home range estimates for the species, we show that Hainan gibbon social groups in the remnant population at Bawangling have larger home ranges than expected in the context of the strong phylogenetic signal that exists for this trait within the Hylobatidae. Our predictive models show that large home range size in gibbons is associated with both intrinsic and extrinsic drivers (low group density in combination with polygynous mating system and larger group size), suggesting that the large Hainan gibbon group sizes at Bawangling may be an artefact of their current reduced population condition; gibbon population density and group density at Bawangling are critically low, so that home ranges of the last remaining social groups may be expanded as a result of a lack of adjacent groups [7].

However, our analyses show that populations of *N. concolor* and *N. nasutus*, the two gibbon species that are most closely related to *N. hainanus* [47], also have larger home range values than expected on the basis of phylogenetic signal alone. These two *Nomascus* species have the largest home ranges of any gibbons other than *N. hainanus*, with home ranges of 1.3–1.5 km^2^, together with large polygynous groups averaging >6 individuals, having been reported in both species [20, 21, 83]. *Nomascus concolor* and *N. nasutus* are also Critically Endangered [53], and have greatly reduced population sizes and densities restricted to limited, isolated and disturbed habitat [84, 85]; low group densities, together with the large polygynous groups observed in both species, may therefore again explain these high home range values. However, other less closely related Critically Endangered gibbon species or isolated gibbon populations persisting at low population densities (e.g. northern white-cheeked gibbon *Nomascus leucogenys*; eastern hoolock gibbon *Hoolock leuconedys* population in Gaoligongshan, China) do not show unexpectedly high home range values in our analyses, and the close phylogenetic relationship between *Nomascus concolor*, *N. hainanus* and *N. nasutus* may alternately indicate a different rate of character evolution in these three species, with large home ranges evolutionarily characteristic for basal *Nomascus* taxa rather than an ecologically abnormal feature exhibited only by reduced populations. Whether quality of available habitat at Bawangling contributes to the large spatial requirements of the remaining Hainan gibbon groups will therefore require further focused investigation; however, the lack of direct correlation of home range with productivity (NDVI), as a metric of site condition, would indicate that actions designed to improve habitat quality alone will be unlikely to address current constraints on Hainan gibbon population recovery. Although we strongly recommend that efforts to protect and enhance available habitat in the Bawangling landscape should be continued and would provide major benefits to the surviving Hainan gibbon population [6–8], more intensive management actions may also be required to enhance the species’ population growth.

In comparison, we show that Hainan gibbon group size, for which there is a strong signal within the Hylobatidae, is no larger than predicted on the basis of phylogeny alone. Furthermore, as hylobatid-wide variation in group size is best explained by species mating system and annual precipitation levels, we find no support for the large size of Hainan gibbon groups being directly linked to any aspects of on-site anthropogenic disturbance at Bawangling. Instead, our results demonstrate flexibility in this behavioural trait across the Hylobatidae in response to both intrinsic and extrinsic factors, but not those immediately associated with recent habitat degradation. Although detection of a relationship between gibbon group size and local habitat quality may require finer-scale data on site condition than it was possible to collect, our results suggest that large, polygynous groups may be the normal social structure for the Hainan gibbon. This has important implications for the conservation management of the population. First, social group size and structure are unlikely to represent reliable indicators of the condition of the surviving Hainan gibbon population, in response to either past human pressures or possible future management scenarios. Second, any potential conservation activities that may be considered in the future, for example translocation of individuals to establish a second population, must take into account the complex, polygynous social structure of the species as an intrinsic component of its biology. This is a crucial new insight that has a direct impact on future Hainan gibbon conservation planning, and demonstrates the wider usefulness of the comparative approach in the conservation toolkit used to inform management of highly threatened species.

## Conclusions

Through employing a comparative approach which incorporates data from multiple populations of all 19 currently recognised gibbon species, we revealed both intrinsic and extrinsic drivers of home range size, social group size and mating system across the Hylobatidae. Home range, group size and mating system are all strongly phylogenetically conserved in gibbons, meaning that more closely related gibbon species resemble each other in terms of these behavioural and ecological traits more than expected by chance. Once these phylogenetic signals are accounted for, variation in these key traits is driven by a combination of social and external factors: variation in gibbon home range size is explained by gibbon group density at a site along with mating system (monogamy versus polygyny) and social group size; gibbon social group size is linked to mean annual rainfall (at the site level) and mating system; and, while no explanatory variables were statistically associated with mating system, gibbon mating system, group size and home range appear to be inherently linked traits, with these factors being important, inter-correlated predictors of each other.

By formally contextualising the Hainan gibbon’s observed behavioural and ecological characteristics within family-wide variation in gibbons, we were also able to determine natural population parameters expected for this Critically Endangered species, compared to those that may be driven by current site conditions experienced by the sole remaining Hainan gibbon population. Our results indicate that remnant Hainan gibbon social groups at Bawangling have larger home ranges than expected in the context of the strong phylogenetic signal across the Hylobatidae, which may be a result of the critically low population density and thus group density at this site. However, current Hainan gibbon group size is no larger than predicted from the pattern of phylogenetic relationships alone, and there is no evidence that the observed mating system (polygyny) is driven by any currently existing external drivers, indicating that large, polygynous groups may be the normal social structure for the Hainan gibbon. Our findings therefore have important and direct implications for Hainan gibbon conservation planning, but also more widely enhance our understanding of gibbon ecology. Our study also demonstrates the usefulness of the comparative approach for informing management of species of conservation concern.

### Availability of supporting data

A copy of the newly compiled hylobatid phylogenetic tree used for tests of phylogenetic signal and predictive modelling in this study is available in LabArchives [https://mynotebook.labarchives.com/share/BMC%2520Evo%2520Bio%2520gibbon%2520tree%2520data/MjMuNHw5NzY3Mi8xOC0yL1RyZWVOb2RlLzM5MzE4MDUwMjN8NTkuNA].

## Additional files

Additional file 1:
**Sources of gibbon comparative data for three response variables (home range, group size, mating system) by species and site (gibbon population).** (DOCX 50 kb) Additional file 2:
**Assessment of effect of phylogenetic uncertainty in the form of alternative tree topologies on phylogenetic signals within the Hylobatidae.** (PDF 770 kb) Additional file 3:
**Global multiple regression linear mixed-effects kinship models incorporating all significant predictors (** ***P*** **< 0.05) from separate single regression models for: a) home range and b) group size.** Significant P-values in bold. (DOCX 19 kb) Additional file 4:
**Ranking of candidate models (representing all possible combinations of the five predictors in each global model) by AICc, along with relative log-likelihood (RLL), ΔAICc, and model Akaike weights (** ***w***_***i***_ **) for: a) home range and b) group size.** (DOCX 25 kb)

## Abbreviations

AIC: Akaike’s information criterion
AICc: Akaike’s information criterion with correction for small sample size
*D*: Phylogenetic signal statistic for binary traits
GHF: Global human footprint data
GS: Group size
HR: Home range
IT: Information theoretic
λ: Pagel’s lambda phylogenetic signal statistic
lmekin: Linear mixed-effects kinship model fit by maximum likelihood
ML: Maximum likelihood
MS: Mating system
mya: Million years ago
NDVI: Normalized difference vegetation index
PGLM: Phylogenetic generalized linear model
RI: Relative importance
RLL: Relative log-likelihood
