# Supplementary material for: Identifying environmental versus phylogenetic correlates of behavioural ecology in gibbons: implications for conservation management of the world’s rarest ape
Source: BMC Evol Biol. 2015 Aug 25;15:171. doi: 10.1186/s12862-015-0430-1 (PMC4549120; doi:10.1186/s12862-015-0430-1)
Supplement: Additional file 1: — Sources of gibbon comparative data for three response variables (home range, group size, mating system) by species and site (gibbon population). (DOCX 50 kb) [file 12862_2015_430_MOESM1_ESM.docx]

# Additional file 1 Sources of gibbon comparative data for three response variables (home range, group size, mating system) by species and site (gibbon population).

| **Species** | **Country** | **Site location** | **Home range reference(s)** | **Group size reference(s)** | **Mating system reference(s)** |
| --- | --- | --- | --- | --- | --- |
| *Hoolock hoolock* | Bangladesh | Lawachara National Park, West Bhanugach Reserve Forest, Maulavibazar | [1],[2] | [1],[2] | [1],[2] |
| *Hoolock hoolock* | India | Bherjan Borajan Padumoni Wildlife Sanctuary, Assam | Jayanta Das (pers. comm., April 2013) | Jayanta Das (pers. comm., April 2013) | Jayanta Das (pers. comm., April 2013) |
| *Hoolock hoolock* | India | Dehing Patkai Wildlife Sanctuary, Assam | Jihosuo Biswas (pers. comm., June 2013) | [3], Jihosuo Biswas (pers. comm., June 2013) | Jihosuo Biswas (pers. comm., June 2013) |
| *Hoolock leuconedys* | India | Mehao Wildlife Sanctuary, Arunachal Pradesh | Jihosuo Biswas (pers. comm., June 2013) | [3], Jihosuo Biswas (pers. comm., June 2013) | Jihosuo Biswas (pers. comm., June 2013) |
| *Hoolock leuconedys* | China | Nankang Park, Gaoligongshan Nature Reserve, Yunnan | [4] | [5] | [3],[5] |
| *Hoolock leuconedys* | Myanmar | Mahamyaing wildlife sanctuary, Sagaing Division | [6] | [6] | [6] |
| *Hylobates abbotti* | Malaysia | Lanjak Entimau Wildlife Sanctuary, Sarawak | [7] | [7] | [3],[7] |
| *Hylobates agilis* | Malaysia | Sungai Dal, Gunong Bubu Forest Reserve, Malay Peninsular | [8],[9] | [8],[10] | [8],[9] |
| *Hylobates agilis* | Indonesia | Kulai Tanang, Kerinci Seblat National Park, Jambi, Sumatra | [11] | [11] | [11] |
| *Hylobates agilis* | Indonesia | Way Canguk Research Area, Bukit Barisan Selatan National Park, Lampung Province | Alice Elder (pers. comm., April 2013) | Alice Elder (pers. comm., April 2013) | Alice Elder (pers. comm., April 2013) |
| *Hylobates albibarbis* | Indonesia | Gunung Palung National Park, West Kalimantan | Andrew Marshall (pers. comm., April 2013) | [12], Andrew Marshall (pers. comm., April 2013) | [3], Andrew Marshall (pers. comm., April 2013) |
| *Hylobates albibarbis* | Indonesia | Sabangau, Central Kalimantan | Susan Cheyne (pers. comm., April 2013) | [13], Susan Cheyne (pers. comm., April 2013) | [3], Susan Cheyne (pers. comm., April 2013) |
| *Hylobates funereus* | Indonesia | Kutai, Kalimantan | [14] | [10] | [14] |
| *Hylobates funereus* | Indonesia | Kayan Mentarang National Park, Kalimantan | [15] | [15] | [3] |
| *Hylobates klossii* | Indonesia | Paitan River area, Siberut Island, West Sumatra | [16] | [3],[10],[16] | [3] |
| *Hylobates klossii* | Indonesia | Pungut Field Station, Peleonan Forest, Siberut, West Sumatra | Marcel Quinten (pers. comm., April 2013) | [17],[18] | Marcel Quinten (pers. comm., April 2013) |
| *Hylobates lar* | Malaysia | Tanjong Triang, Johore | [10] | [10] | [19] |
| *Hylobates lar* | Malaysia | Kuala Lompat, Krau Game Reserve, Pahang | [14] | [10] | [20] |
| *Hylobates lar* | Thailand | Khlong Sai study site, Khao Yai National Park, Nakhon Nayok | [21] | Warren Brockelman (pers. comm., June 2013) | [21] |
| *Hylobates lar* | Thailand | Mo Singto site, Khao Yai National Park, Nakhon Nayok | [22] | Warren Brockelman (pers. comm., June 2013), Ulrich Reichard (pers. comm., April 2013) | [3],[23], Warren Brockelman (pers. comm., June 2013) |
| *Hylobates moloch* | Indonesia | Gunung Halimun-Salak National Park, West Java | [24] | [24] | Sanha Kim (pers. comm., April 2013) |
| *Hylobates moloch* | Indonesia | Cagar Alam Leuweung Sancang Nature Reserve, Java | [25],[26] | [25],[26] | [26] |
| *Hylobates moloch* | Indonesia | Turalak, Ujung Kulou, Java | [14],[27] | [14],[27] | [3] |
| *Hylobates moloch* | Indonesia | Sokokembang forest, Central Java | Arif Setiawan (pers. comm., April 2013) | Arif Setiawan (pers. comm., April 2013) | Arif Setiawan (pers. comm., April 2013) |
| *Hylobates muelleri* | Indonesia | Sungai Wain Protection Forest, Balikpapan, East Kalimantan | Vincent Nijman (pers. comm., April 2013) | [28], Vincent Nijman (pers. comm., April 2013) | Susan Cheyne (pers. comm., April 2013), Vincent Nijman (pers. comm., April 2013) |
| *Hylobates pileatus* | Thailand | Khlong Sai study site, Khao Yai National Park, Nakhon Nayok | [21] | Warren Brockelman (pers. comm., June 2013) | [3],[21] |
| *Hylobates pileatus* | Thailand | Khao Ang Rue Nai Wildlife Sanctuary, Chachoengsao Province | Rungnapa Phoonjampa (pers. comm., April 2013) | [29], Rungnapa Phoonjampa (pers. comm., April 2013) | Rungnapa Phoonjampa (pers. comm., April 2013) |
| *Hylobates pileatus* | Thailand | Khao Soi Dai, Khao Khitchakut District | [30] | [10],[31] | [23],[30] |
| *Nomascus annamensis* | Cambodia | Veun Sai-Siem Pang Conservation Area, Stung Treng and Ratanakiri Province | Ben Rawson (pers. comm., April 2013) | Ben Rawson (pers. comm., April 2013) | Ben Rawson (pers. comm., April 2013) |
| *Nomascus concolor* | China | Dazhaizi, Wuliang Mt., Jingdong County, Yunnan | [32],[33], Fan Peng Fei (pers. comm., April 2013) | [34], Fan Peng Fei (pers. comm., April 2012) | [32],[33],[35], Fan Peng Fei (pers. comm., April 2013) |
| *Nomascus concolor* | China | Xiaobahe, Wuliang Mt., Zhenyuan County, Yunnan | [36] | [36],[37] | [35],[36],[38-40] |
| *Nomascus gabriellae* | Vietnam | Cat Tien National Park, Dong Nao | Marina Kenyon (pers. comm., April 2013) | Marina Kenyon (pers. comm., April 2013) | Marina Kenyon (pers. comm., April 2013) |
| *Nomascus hainanus* | China | Bawangling National Nature Reserve, Baisha and Changjiang, Hainan | [41],[42] | [41-43] | [40-42],[44-46] |
| *Nomascus leucogenys* | Laos | Nam Kading National Protected Area, Bolikhamxay Province | Julia Ruppell (pers. comm., April 2013 ) | Julia Ruppell (pers. comm., April 2013) | Julia Ruppell (pers. comm., April 2013) |
| *Nomascus nasutus* | China | Bangliang, Jingxi County, Guangxi | [3],[47], Fan Peng Fei (pers. comm., April 2013) | Fan Peng Fei (pers. comm., April 2013) | [48], Fan Peng Fei (pers. comm., April 2013) |
| *Nomascus siki* | Laos | Nam Kading National Protected Area, Bolikhamxay Province | [7] | Chris Hallam (pers. comm., April 2013) | Chris Hallam (pers. comm., April 2013) |
| *Symphalangus syndactylus* | Malaysia | Kuala Lompat, Krau Game Reserve, Pahang | [20] | [49] | [49] |
| *Symphalangus syndactylus* | Indonesia | Kulai Tanang, Kerinci Seblat National Park, Jambi, Sumatra | [11] | [11] | [11] |
| *Symphalangus syndactylus* | Indonesia | Way Canguk Research Station, Lampung Province, Sumatra | Susan Lappan (pers. comm., April 2013) | [50], Susan Lappan (pers. comm., April 2013) | Tim O'Brien (pers. comm., April 2013), Susan Lappan (pers. comm., April 2013) |

## References

1. Islam MA, Feeroz MM: **Ecology of hoolock gibbon of Bangladesh.** *Primates.* 1992; **33:**451-64.
2. Österberg P: **The vanishing ape of Bangladesh: a report from the hoolock gibbon’s last stronghold in the country.** *Gibbon Journal.* 2007; **3:**35-42.
3. Chivers DJ, Anandam MV, Groves CP, Molur S, Rawson BM, Richardson MC, *et al*.: **Family Hylobatidae (gibbons).** In *Handbook of the Mammals of the World. Volume 3. Primates*. Edited by Mittermeier RA, Rylands AB, Wilson DE. Barcelona, Lynx Edicions; 2013:754-91.
4. Zhang D, Fei HL, Yuan SD, Sun WM, Ni QY, Cui LW, *et al*.: **Ranging behavior of eastern hoolock gibbon (*Hoolock leuconedys*) in a northern montane forest in Gaoligongshan, Yunnan, China.** *Primates.* 2014; **55:**239-47.
5. Fan P, Xiao W, Huo S, Ai H, Wang T, Lin R: **Distribution and conservation status of the Vulnerable eastern hoolock gibbon *Hoolock leuconedys* in China.** *Oryx.* 2011; **45:**129-34.
6. Brockelman W, Naing H, Saw C, Moe A, Linn Z, Moe T, *et al*.: **Census of eastern hoolock gibbons (*Hoolock leuconedys*) in Mahamyaing Wildlife Sanctuary, Sagaing Division, Myanmar.** In *The Gibbons: New Perspectives on Small Ape Socioecology and Population Biology.* Edited by Lappan S, Whittaker DJ. New York, Springer; 2009:435-51.
7. Rowe N, Myers M: *All the World’s Primates*. <[www.alltheworldsprimates.org](http://www.alltheworldsprimates.org)> Charlestown, Primate Conservation Inc.; 2011.
8. Gittins SP: **Territorial behavior in the agile gibbon.** *Int J Primatol.* 1980; **1:**381-99.
9. Gittins SP: **Feeding and ranging in the agile gibbon.** *Folia Primatol.* 1982; **38:**39-71.
10. Leighton DR: **Gibbons: territoriality and monogamy.** In *Primate Societies*. Edited by Smuts BB, Cheney DL, Seyfarth RM, Wrangham RW, Struhsaker TT. Chicago and London, University of Chicago Press; 1987:135-45.
11. Yanuar A: *Effects of fragmentation on Siamang Symphalangus syndactylus and Agile gibbon Hylobates agilis in west-central Sumatra*. PhD thesis, University of Cambridge; 2007.
12. Marshall AJ, Cannon CH, Leighton M: **Competition and niche overlap between gibbons (*Hylobates albibarbis*) and other frugivorous vertebrates in Gunung Palung National Park, West Kalimantan, Indonesia.** In *The Gibbons: New Perspectives on Small Ape Socioecology and Population Biology.* Edited by Whittaker D, Lappan S. New York, Springer; 2009:161-88.
13. Hamard M, Cheyne SM, Nijman V: **Vegetation correlates of gibbon density in the peat-swamp forest of the Sabangau catchment, Central Kalimantan, Indonesia.** *Am J Primatol.* 2010; **72:**607-16.
14. Chivers DJ: **Feeding and ranging in gibbons: a summary.** In *The Lesser Apes: Evolutionary and Behavioural Biology* Edited by Preuschoft H, Chivers DJ, Brockelman W, Creel N. Edinburgh, Edinburgh University Press; 1984:267-81.
15. Nijman V, Menken S: **Assessment of census techniques for estimating density and biomass of gibbons (Primates: Hylobatidae).** *Raffles B Zool.* 2005; **53:**169-79.
16. Whitten AJ: **Home range use by Kloss gibbons (*Hylobates klossii*) on Siberut Island, Indonesia.** *Anim Behav* **30:**182-98; 1982.
17. Quinten MC, Syamsuri F, Hodges JK: **Peat swamp forest supports high primate densities on Siberut Island, Sumatra, Indonesia.** *Oryx.* 2009; **44:**147-51.
18. Höing A, Quinten MC, Indrawati YM, Cheyne SM, Waltert M: **Line transect and triangulation surveys provide reliable estimates of the density of Kloss’ gibbons (*Hylobates klossii*) on Siberut Island, Indonesia.** *Int J Primatol.* 2013; **34:**148-56.
19. Ellefson JO: **A natural history of white-handed gibbons in the Malayan Peninsular.** In *Gibbon and Siamang, Vol. 3, Natural history, Social behavior, Reproduction, Vocalizations, Prehension*. Edited by Rumbaugh DM. Basel, Karger; 1974:1-136.
20. Raemaekers J: **Ecology of sympatric gibbons.** *Folia Primatol.* 1979; **31:**227-45.
21. Suwanvecho U, Brockelman W: **Interspecific territoriality in gibbons (*Hylobates lar* and *H. pileatus*) and its effects on the dynamics of interspecies contact zones.** *Primates.* 2012; **53:**97-108.
22. Bartlett TQ: **Seasonal home range use and defendability in white-handed gibbons (*Hylobates lar*) in Khao Yai National Park, Thailand.** In *The Gibbons: New Perspectives on Small Ape Socioecology and Population Biology.* Edited by Whittaker D, Lappan S. New York, Springer; 2009:265-75.
23. Brockelman W, Srikosamatara S: **Maintenance and evolution of social structure in gibbons.** In *The Lesser Apes: Evolutionary and Behavioural Biology*. Edited by Preuschoft H, Chivers DJ, Brockelman W, Creel N. Edinburgh, Edinburgh University Press; 1984:298-323.
24. Kim S, Lappan S, Choe JC: **Diet and ranging behavior of the endangered Javan gibbon (*Hylobates moloch*) in a submontane tropical rainforest.** *Am J Primatol.* 2011; **73:**270-80.
25. Malone N: *The socioecology of the Critically Endangered Javan gibbon (Hylobates moloch): assessing the impact of anthropogenic disturbance on primate social systems*. PhD thesis, University of Oregon; 2007.
26. Malone N, Fuentes A: **The ecology and evolution of hylobatid communities: causal and contextual factors underlying inter- and intraspecific variation.** In *The Gibbons: New Perspectives on Small Ape Socioecology and Population Biology.* Edited by Whittaker D, Lappan S. New York, Springer; 2009:241-64.
27. Kappeler M: **Vocal bouts and territorial maintenance in the moloch gibbon.** In *The Lesser Apes: Evolutionary and Behavioural Biology*. Edited by Preuschoft H, Chivers DJ, Brockelman W, Creel N. Edinburgh, Edinburgh University Press; 1984:376-89.
28. Gilhooly LJ: *Population Density and Habitat Assessment of Müller’s Gibbon (Hylobates muelleri) in Sungai Wain Protection Forest, East Kalimantan, Indonesia*. MSc thesis, Oxford Brookes University; 2012.
29. Phoonjampa R, Koenig A, Brockelman W, Borries C, Gale G, Carroll J, *et al.*: **Pileated gibbon density in relation to habitat characteristics and post‐logging forest recovery.** *Biotropica.* 2011; **43:**619-27.
30. Srikosamatara S, Brockelman W: **Polygyny in a group of pileated gibbons via a familial route.** *Int J Primatol.* 1987; **8:**389-93.
31. Brockelman W, Srikosamatara S: **Estimation of density of gibbon groups by use of loud songs.** *Am J Primatol.* 1993; **29:**93-108.
32. Fan P, Jiang X: **Effects of food and topography on ranging behavior of black crested gibbon (*Nomascus concolor jingdongensis*) in Wuliang Mountain, Yunnan, China.** *Am J Primatol.* 2008; **70:**871-8.
33. Fan P, Jiang, X: **Sleeping sites, sleeping trees, and sleep-related behaviors of black crested gibbons (*Nomascus concolor jingdongensis*) at Mt. Wuliang, Central Yunnan, China.** *Am J Primatol.* 2008; **70:**153-60.
34. Fan P, Jiang X: **Maintenance of multifemale social organization in a group of *Nomascus concolor* at Wuliang Mountain, Yunnan, China.** *Int J Primatol.* 2010; **31:**1-13.
35. Fan P, Jiang X, Liu C, Luo W: **Polygynous mating system and behavioural reason of black crested gibbon (*Nomascus concolor jingdongensis*) at Dazhaizi, Mt. Wuliang, Yunnan, China.** *Zool Res.* 2006; **27:**216-20.
36. Jiang X, Wang Y: **Popluation and conservation of black-crested gibbons (*Hylobates concolor jingdongensis*) in Wuliang Nature Reserve, Jingdong, Yunnan.** *Zool Res.* 1999; **20:**421-5.
37. Sheeran LK: *A preliminary study of the behavior and socio-ecology of Black gibbons (Hylobates concolor) in Yunnan Province, People's Republic of China*. PhD thesis, Ohio State University Ohio; 1993.
38. Haimoff EH, Yang XJ, He SJ, Chen N: **Census and survey of wild black-crested gibbons (*Hylobates concolor concolor*) in Yunnan Province, People's Republic of China.** *Folia Primatol.* 1986; **46:**205-14.
39. Haimoff EH, Yang XJ, He SJ, Chen N: **Preliminary observations of wild black-crested gibbons (*Hylobates concolor concolor*) in Yunnan province, people's republic of China.** *Primates.* 1987; **28:**319-35.
40. Bleisch WV, Chen N: **Ecology and behavior of wild black-crested gibbons (*Hylobates concolor*) in China with a reconsideration of evidence for polygyny.** *Primates.* 1991; **32:**539-48.
41. Bryant JV: *Developing a Conservation Evidence-Base for the Critically Endangered Hainan Gibbon (Nomascus hainanus)*. PhD thesis, University College London; 2014.
42. Zhou J, Wei F, Li M, Pui Lok C, Wang D: **Reproductive characters and mating behaviour of wild *Nomascus hainanus*.** *Int J Primatol.* 2008; **29:**1037-46.
43. Zhou J, Chan BPL, Wei F: **Responses to inter-group encounters of the Hainan gibbon *Nomascus hainanus*.** *Zool Res.* 2008; **29:**667-73.
44. Liu Z, Jiang H, Zhang Y, Liu Y, Chou T, Manry D, *et al.*: **Field report on the Hainan gibbon.** *Primate Conserv.* 1987; **8:**49-50.
45. Liu Z, Zhang Y, Jiang H, Southwick C: **Population structure of *Hylobates concolor* in Bawanglin Nature Reserve, Hainan, China.** *Am J Primatol.* 1989; **19:**247-54.
46. Wu W, Wang X, Claro F, Ding Y, Souris AC, Wang C, *et al*.: **The current status of the Hainan black-crested gibbon *Nomascus* sp. cf. *nasutus hainanus* in Bawangling National Nature Reserve, Hainan, China.** *Oryx.* 2004; **38:**452-6.
47. Fei H, Scott MB, Zhang W, Ma C, Xiang Z, Fan P: **Sleeping tree selection of Cao Vit gibbon (*Nomascus nasutus*) living in degraded karst forest in Bangliang, Jiangxi, China.** *Am J Primatol.* 2012; **74:**998-1005.
48. Fan P, Fei H, Xiang Z, Zhang W, Ma C, Huang T: **Social Structure and Group Dynamics of the Cao Vit Gibbon (*Nomascus nasutus*) in Bangliang, Jingxi, China.** *Folia Primatol.* 2010; **81:** 245-53.
49. Gittins SP, Raemaekers J: **Siamang, lar and agile gibbons.** In *Malayan Forest Primates: Ten Years' Study in Tropical Rain Forest*. Edited by Chivers DJ. New York, Plenum Press; 1980:63-105.
50. O'Brien T, Kinnaird M, Nurcahyo A, Iqbal M, Rusmanto M: **Abundance and distribution of sympatric gibbons in a threatened Sumatran rain forest.** *Int J Primatol.* 2004; **25:**267-84.
