# Supplementary material for: Identifying environmental versus phylogenetic correlates of behavioural ecology in gibbons: implications for conservation management of the world’s rarest ape
Source: BMC Evol Biol. 2015 Aug 25;15:171. doi: 10.1186/s12862-015-0430-1 (PMC4549120; doi:10.1186/s12862-015-0430-1)
Supplement: Additional file 2: — Assessment of effect of phylogenetic uncertainty in the form of alternative tree topologies on phylogenetic signals within the Hylobatidae. (PDF 770 kb) [file 12862_2015_430_MOESM2_ESM.pdf]

Appendix S2. Assessment of effect of phylogenetic uncertainty in the form of alternative tree topologies on phylogenetic signals within the Hylobatidae.

Due to the apparent difficulty of resolving the pattern of phylogenetic branching within the Hylobatidae [1],[2], limitations associated with the most complete gibbon phylogenetic tree available in terms of species assemblage (single locus data used to construct the phylogeny; some nodes of tree are poorly supported) [3], and concerns regarding the sensitivity of comparative analyses to the choice of phylogenetic tree [4],[5], we performed a preliminary investigation to explore the impact of phylogenetic uncertainty upon our principal comparative analysis testing phylogenetic signal. Using a range of alternative phylogenetic tree topologies drawn from the available literature, each representing different possible relationships among gibbons, we tested for the presence and strength of any phylogenetic signal in the traits of interest, including: body mass (as a control); home range; group size; and mating system.

An additional five published phylogenetic trees [6-10] incorporating gibbons and the two *Pongo* species (as an outgroup) were tested, in addition to the published tree of Thinh *et al.* (2010) [3] and our *a priori* preferred tree, a modified version of this tree also including *N. annamensis* (see Figures S1-7). These alternative trees differed both in the number of gibbon species incorporated (and generally did not include the main species of interest, *Nomascus hainanus*) and in the topology of relationships between different species, but all of these trees included ≥8 gibbon species (i.e. they represented at least half of all gibbon species recognized before the recent establishment of three new species, *Hylobates abbotti*, *H. funereus* [11], and *Nomascus annamensis* [12]. This set of seven possible trees included phylogenies

derived from a variety of methodological approaches, including several trees that used multiple genetic markers/regions (e.g. [8],[9]).

We tested for phylogenetic signal across the seven alternative trees using Pagel's lambda (λ) [13],[14] for continuous traits and *D* [15],[16] for categorical traits and using high and low estimates for Hainan gibbon home range [17],[18], as described in the main methods section. The frequency distribution of resultant λ and *D* signal values was then plotted to investigate variation in the strength of the phylogenetic signal in each trait when alternative phylogenetic trees were utilized. For *D*, only six trees in total returned signals due to the absence of *Nomascus* from one tree [6] which prevented variation in this trait being captured and thus adequate representation of this binary-coded variable in this instance.

Our results of phylogenetic signal in all four traits were consistent, despite the phylogenetic uncertainty introduced by testing alternative gibbon species assemblages and relationships across the different tree topologies. We observed very similar values of λ for body mass, home range (for both high and low Hainan gibbon estimates), and group size across all trees tested, with tight clustering of signals around λ=1 (Figures S8-11), supporting strong patterns of phylogenetic signal in each of these traits. Similarly, values of *D* for mating system varied from -0.5 to -2.0 but clustered around -1.0 to -1.5 (Figure S12), again indicating strong phylogenetic conservatism in this trait. The strength of the phylogenetic signals observed across all trees for the three continuous traits was consistent ($\lambda \geq 0.82$), as was the direction (negative) and magnitude of the signal observed for the categorical trait ($D \leq -0.54$). This consistency indicates that the choice of tree topology in these cases is not critical to the phylogenetic signals observed; indeed it appears to have little impact on these signals, apparently because the signals are so strong. The signals returned for each

trait by our *a priori* preferred phylogeny (indicated by the red lines in Figures S8-12) align with the most frequently observed signal value for all four traits, indicating that use of this preferred tree alone for all subsequent comparative analyses within this study is valid, and will not reduce the power, reliability, or relevance of results of any of these analyses.

References

1. Carbone L, Harris RA, Gnerre S, Veeramah KR, Lorente-Galdos B, Huddleston J, *et al.*: **Gibbon genome and the fast karyotype evolution of small apes.** *Nature.* 2014; **513:**195-201.
2. Veeramah KR, Woerner AE, Johnstone L, Gut I, Gut M, Marques-Bonet T, *et al.*: **Examining phylogenetic relationships among gibbon genera using whole genome sequence data using an approximate Bayesian computation approach.** *Genetics.* 2015; doi:10.1534/genetics.115.174425.
3. Thinh VN, Mootnick A, Geissmann T, Li M, Ziegler T, Agil M, *et al.*: **Mitochondrial evidence for multiple radiations in the evolutionary history of small apes.** *BMC Evol Biol.* 2010; **10:**74.
4. Arnold C, Matthews LJ, Nunn CL: **The 10kTrees website: a new online resource for primate phylogeny**. *Evol Anthropol*. 2010; **19:**114-18.
5. Pozzi L, Bergey CM, Burrell AS: **The use (and misuse) of phylogenetic trees in comparative behavioral analyses**. *Int J Primatol*. 2014; **35:**32-54.
6. Bininda-Emonds ORP, Cardillo M, Jones K, MacPhee R, Beck R, Grenyer R, *et al.*: **The delayed rise of present-day mammals**. *Nature*. 2007; **446:**507-12.

7. Chan Y, Roos C, Inoue-Murayama M, Inoue E, Shih C, Pei K, *et al.*: **Mitochondrial genome sequences effectively reveal the phylogeny of Hylobates gibbons**. *PLoS One.* 2010; **5:**e14419.
8. Faurby S, Svenning JC: **A species-level phylogeny of all extant and late Quaternary extinct mammals using a novel heuristic-hierarchical Bayesian approach**. *Mol Phylogenet Evol.* 2015; **84:**14-26.
9. Perelman P, Johnson WE, Roos C, Seuánez HN, Horvath JE, Moreira MA, *et al.*: **A molecular phylogeny of living primates**. *PLoS Genet.* 2011; **7:**e1001342.
10. Springer MS, Meredith RW, Gatesy J, Emerling CA, Park J, Rabosky DL, *et al.*: **Macroevolutionary dynamics and historical biogeography of primate diversification inferred from a species supermatrix.** *PloS One.* 2012; **7:**e49521.
11. Chivers, DJ, Anandam MV, Groves CP, Molur S, Rawson BM, Richardson MC, *et al.*: **Family Hylobatidae (gibbons).** In *Handbook of the Mammals of the World. Volume 3. Primates*. Edited by Mittermeier RA, Rylands AB, Wilson DE. Barcelona, Lynx Edicions; 2013:754-91.
12. Thinh VN, Mootnick A, Thanh VN, Nadler T, Roos C: **A new species of crested gibbon, from the central Annamite mountain range.** *Viet J Primatol.* 2010; **1:**1-12.
13. Pagel M: **Inferring the historical patterns of biological evolution.** *Nature.* 1999; **401:**877-84.
14. Freckleton RP, Harvey PH, Pagel M: **Phylogenetic analysis and comparative data: a test and review of evidence.** *Am Nat.* 2002; **160:**712-26.

15. Fritz SA, Purvis A: **Selectivity in mammalian extinction risk and threat types: A new measure of phylogenetic signal strength in binary traits.** *Cons Biol.* 2010; **24:**1042-51.
16. Orme D, Freckleton R, Thomas G, Petzoldt T, Fritz S, Isaac N, *et al.*: *caper: Comparative Analyses of Phylogenetics and Evolution in R.* R Package Version 0.5.2; 2013.
17. Zhou J, Wei F, Li M, Pui Lok C, Wang D: **Reproductive characters and mating behaviour of wild *Nomascus hainanus*.** *Int J Primatol.* 2008; **29:**1037-46.
18. Bryant JV: *Developing a Conservation Evidence-Base for the Critically Endangered Hainan Gibbon (Nomascus hainanus).* PhD thesis, University College London; 2014.

Figures S1-7 **Phylogenetic uncertainty:** Seven alternative phylogenetic trees representing possible relationships among gibbons used for tests of phylogenetic signal in four traits (body mass, home range group size, and mating system) across the Hylobatidae. Trees standardised as chronograms, scales indicate branch length in million years before present (MYA).

Figures S8-11 **Effect of phylogenetic uncertainty on phylogenetic signal in continuous traits:** Histograms showing the frequency distribution of values of Pagel's lambda (λ) from the tests of phylogenetic signal using seven different phylogenetic tree topologies for the continuous traits of body mass, home range (using two estimates of Hainan gibbon home range), and group size. The red line indicates the signal (λ) returned by our *a priori* preferred phylogeny.

Figure S12 **Effect of phylogenetic uncertainty on phylogenetic signal in a** categorical trait: Histogram showing the frequency distribution of values of *D* from the tests of phylogenetic signal using six different phylogenetic tree topologies for the categorical trait of mating system. The red line indicates the signal (*D*) returned by our *a priori* preferred phylogeny.

Bininda−Emonds *et al.* 2007

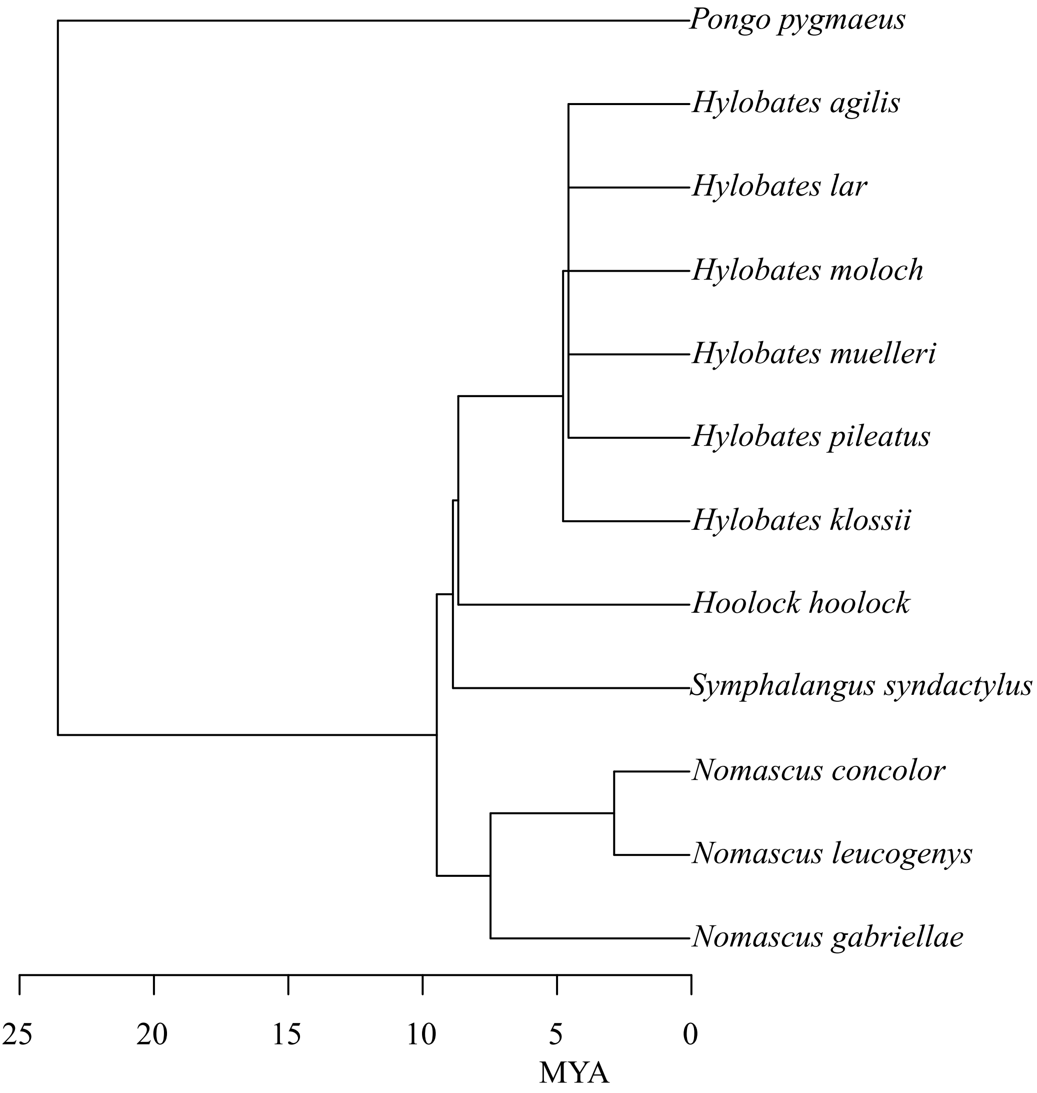

Chan *et al.* 2010

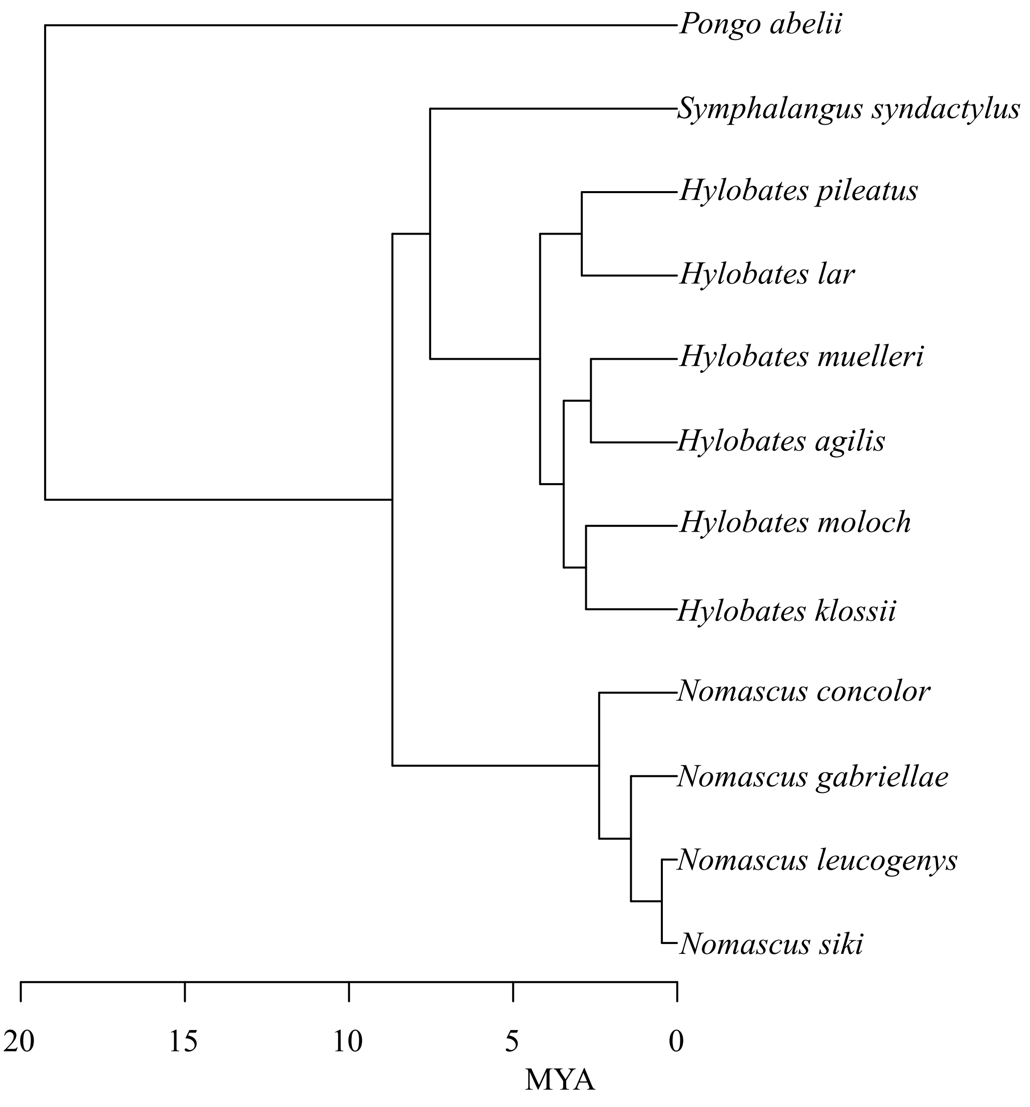

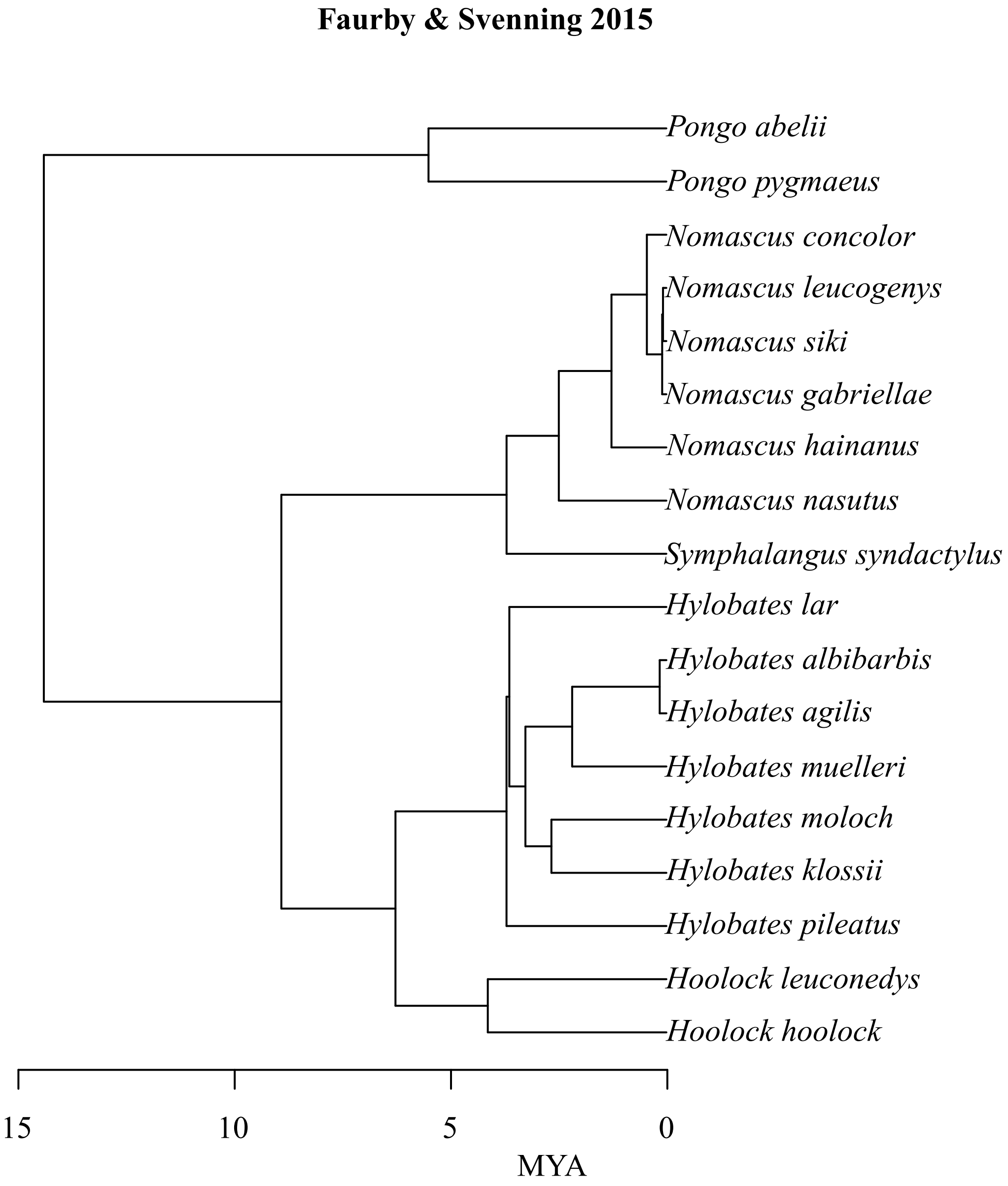
Faurby & Svenning 2015
Pongo abelii
Pongo pygmaeus
Nomascus concolor
Nomascus leucogenys
Nomascus siki
Nomascus gabriellae
Nomascus hainanus
Nomascus nasutus
Symphalangus syndactylus
Hylobates lar
Hylobates albibarbis
Hylobates agilis
Hylobates muelleri
Hylobates moloch
Hylobates klossii
Hylobates pileatus
Hoolock leuconedys
Hoolock hoolock
15
10
5
0
MYA

Perelman *et al.* 2011

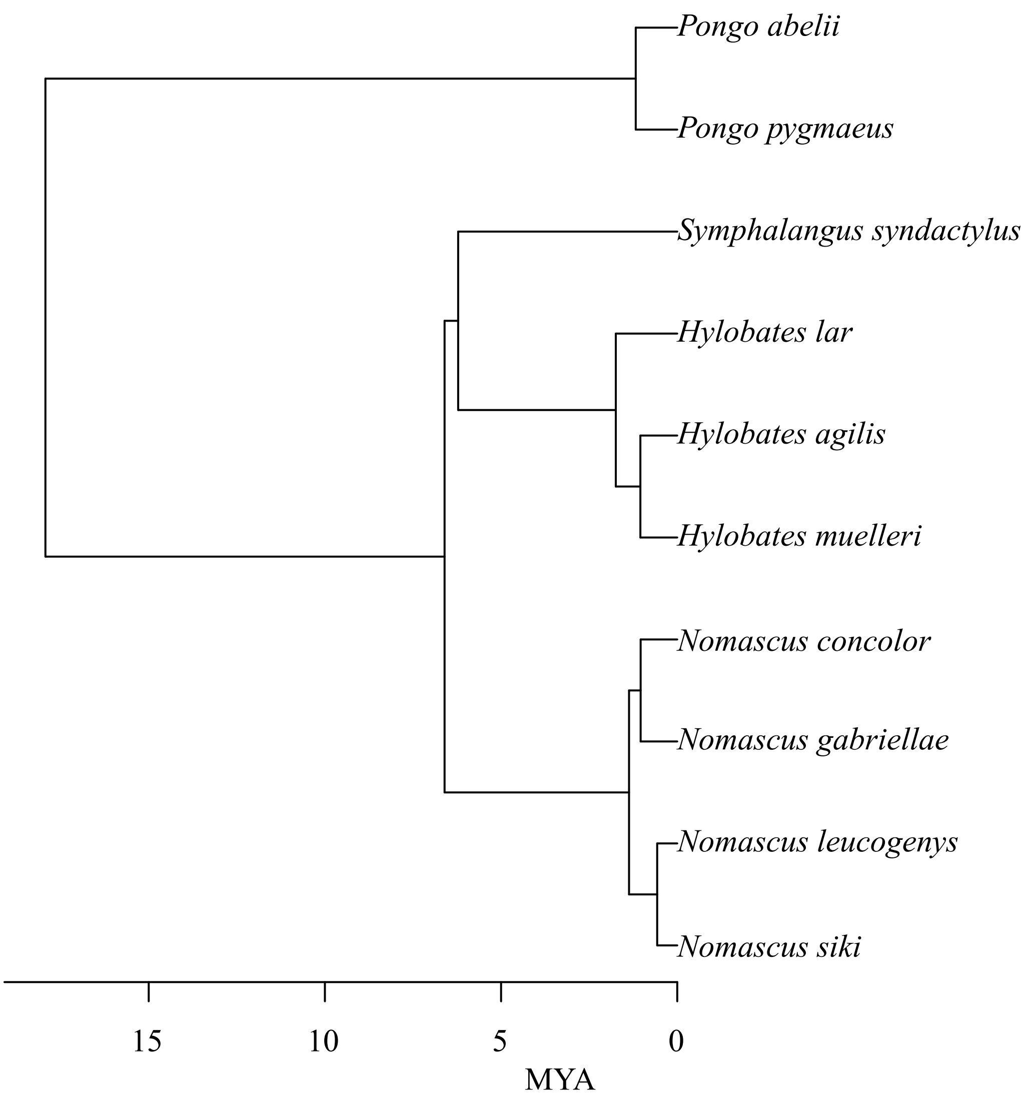

Springer *et al.* 2012

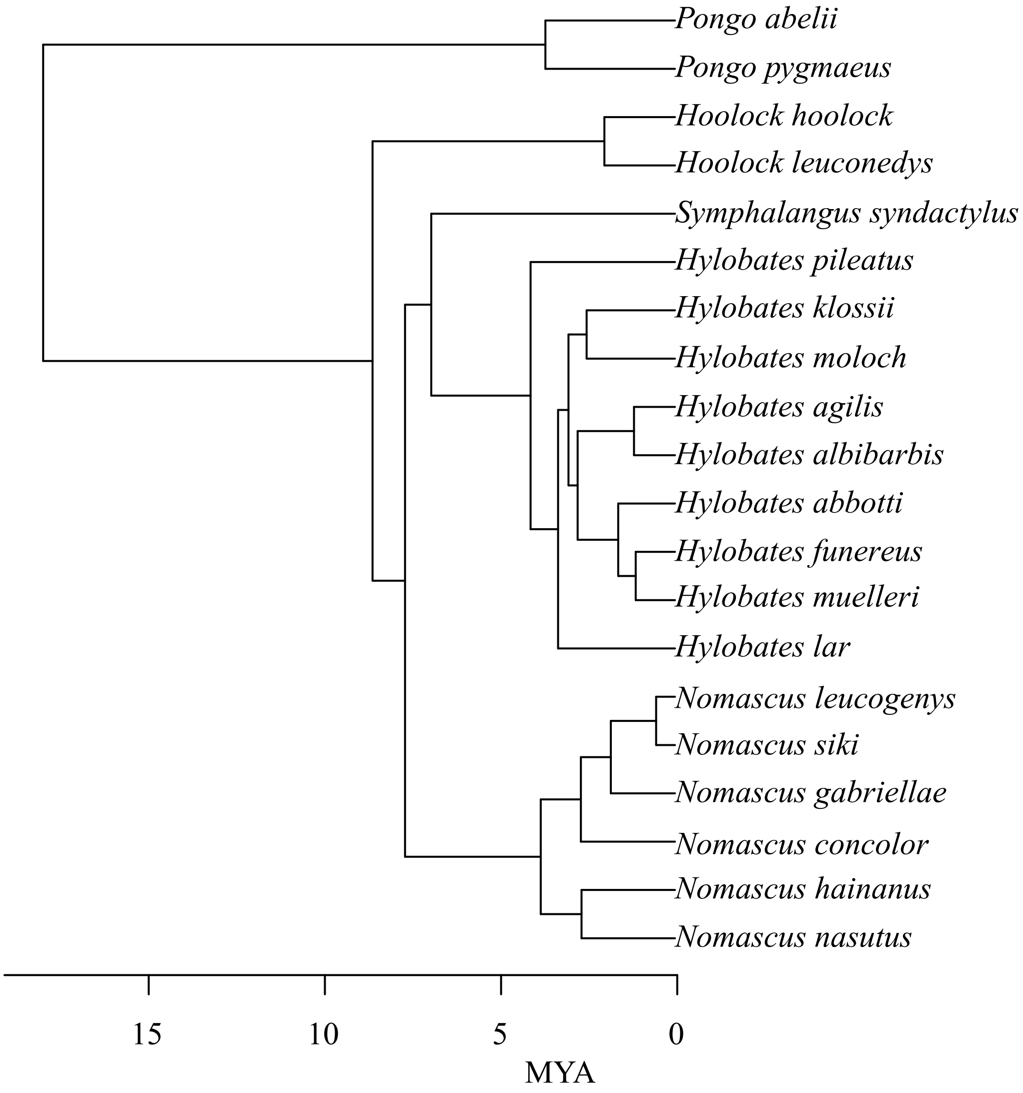

Thinh *et al.* 2010

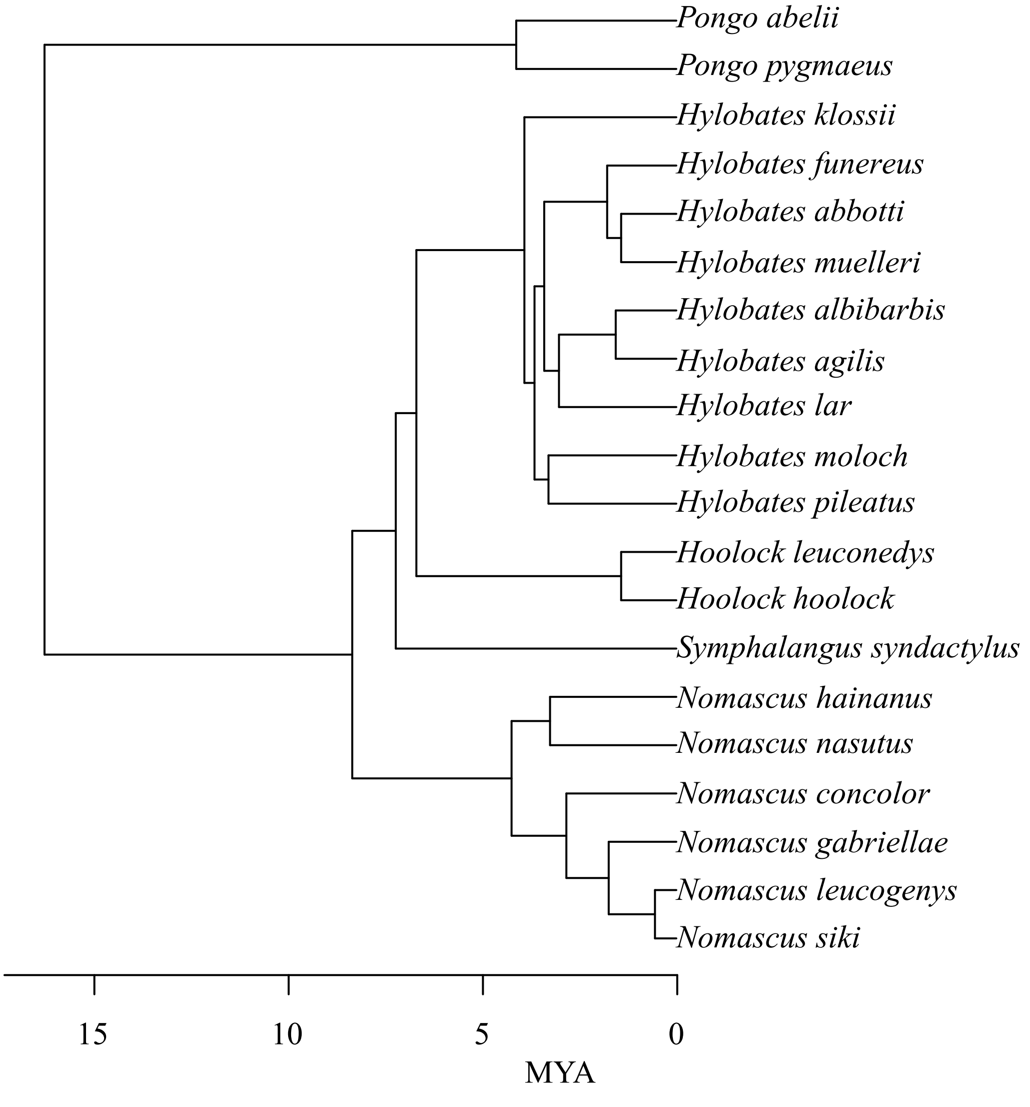

Thinh *et al.* 2010 edited (*Nomascus annamensis* added)

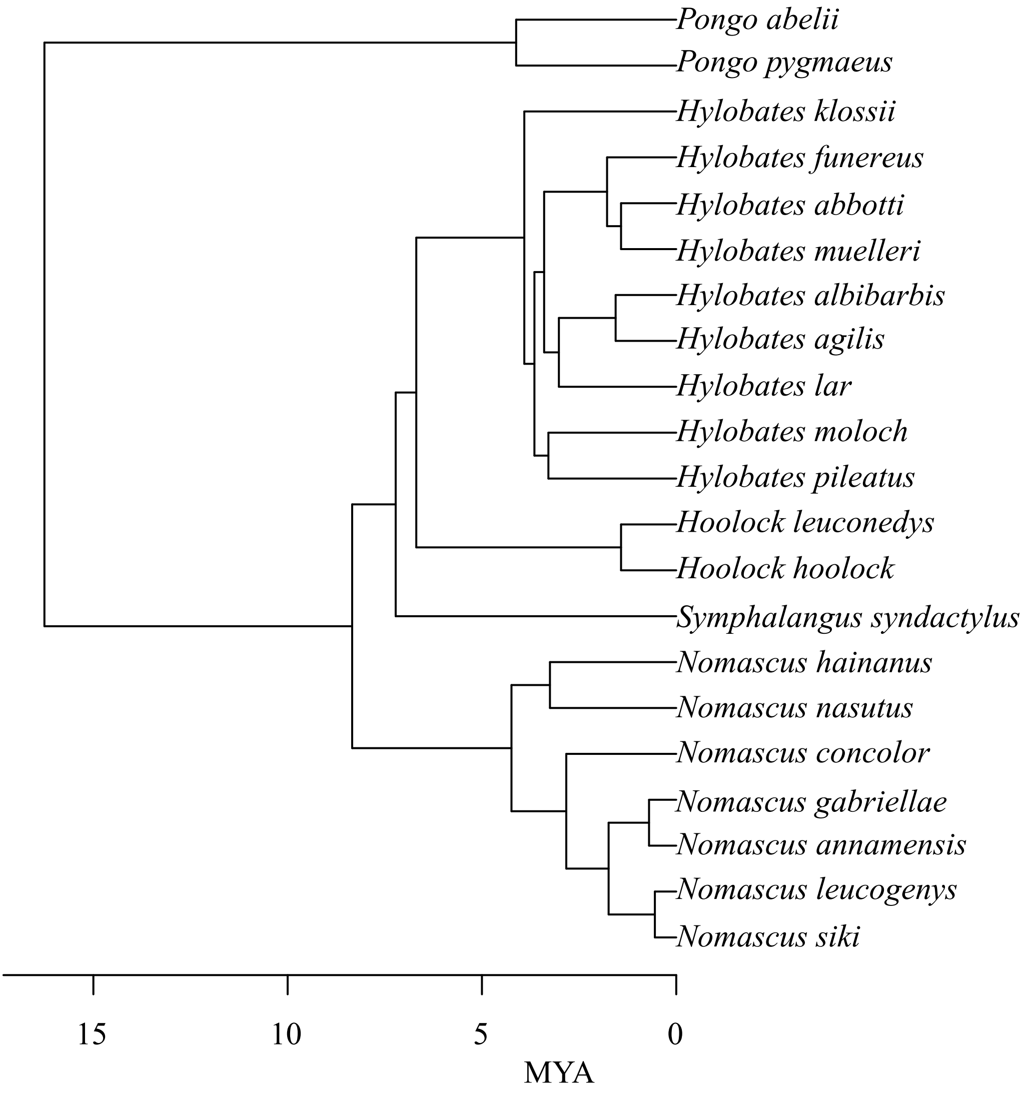

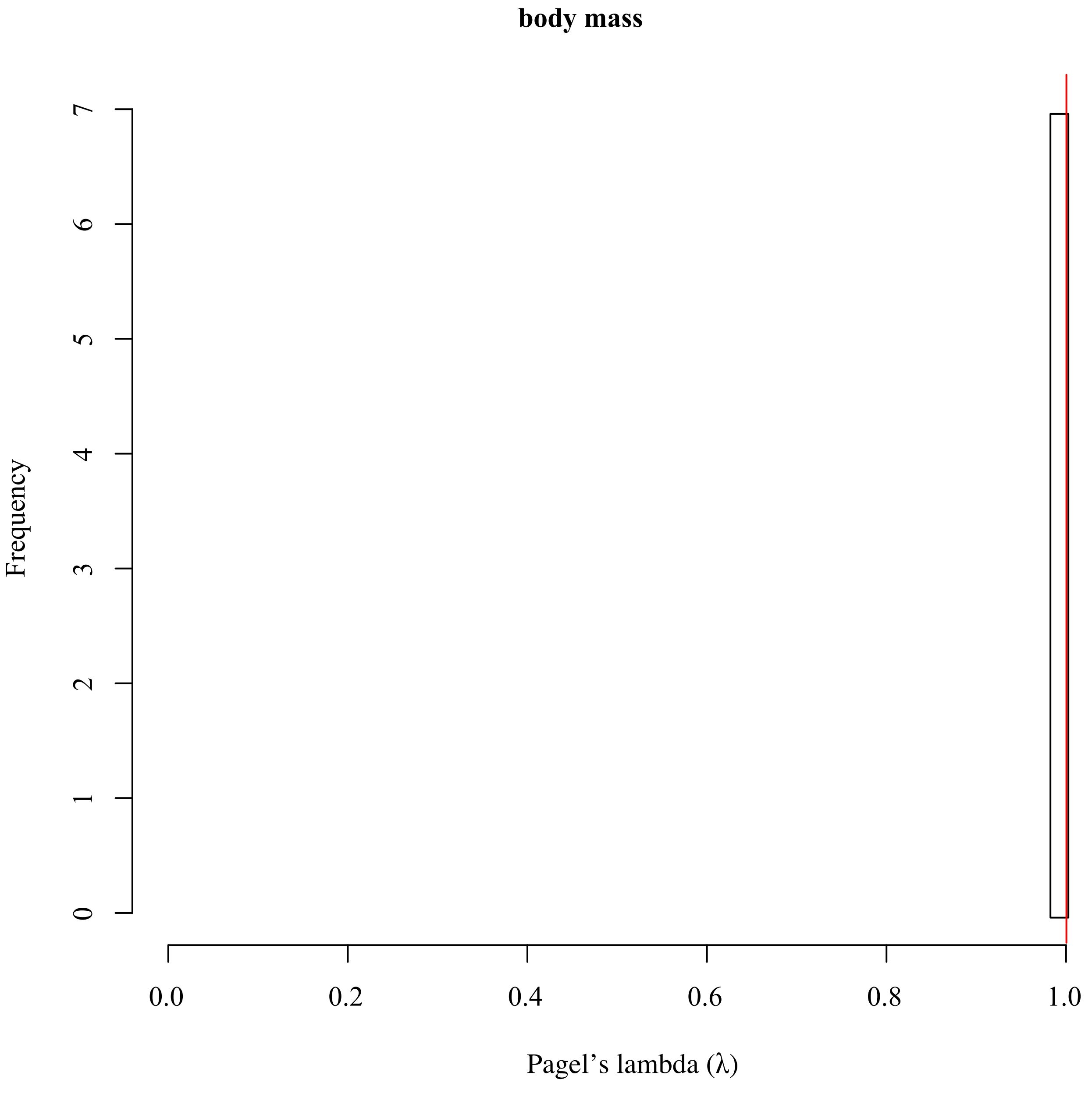
body mass
Frequency
0
1
2
3
4
5
6
7
0.0
0.2
0.4
0.6
0.8
1.0
Pagel's lambda (λ)

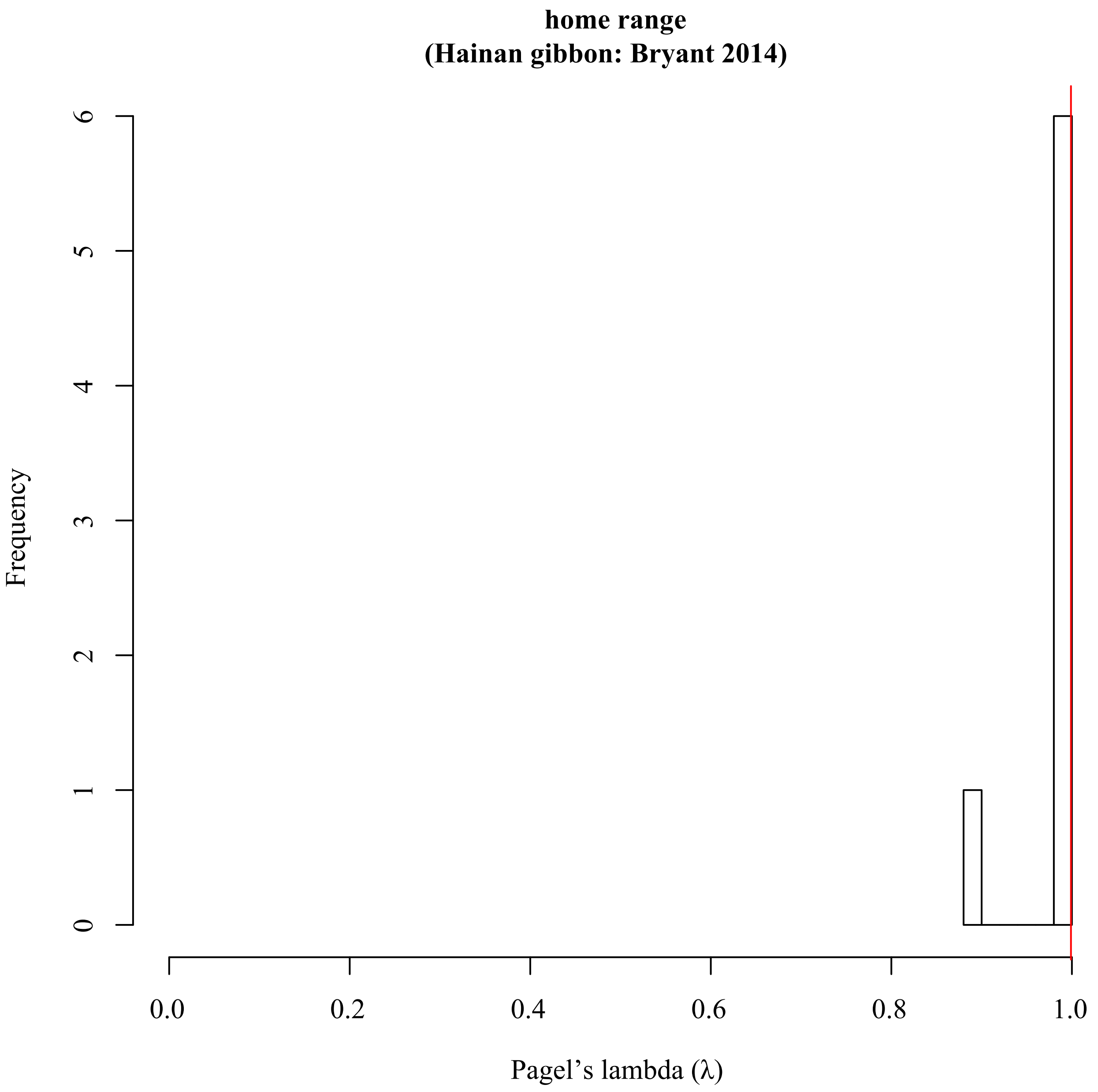
home range
(Hainan gibbon: Bryant 2014)
Frequency
0
1
2
3
4
5
6
0.0
0.2
0.4
0.6
0.8
1.0
Pagel's lambda (λ)

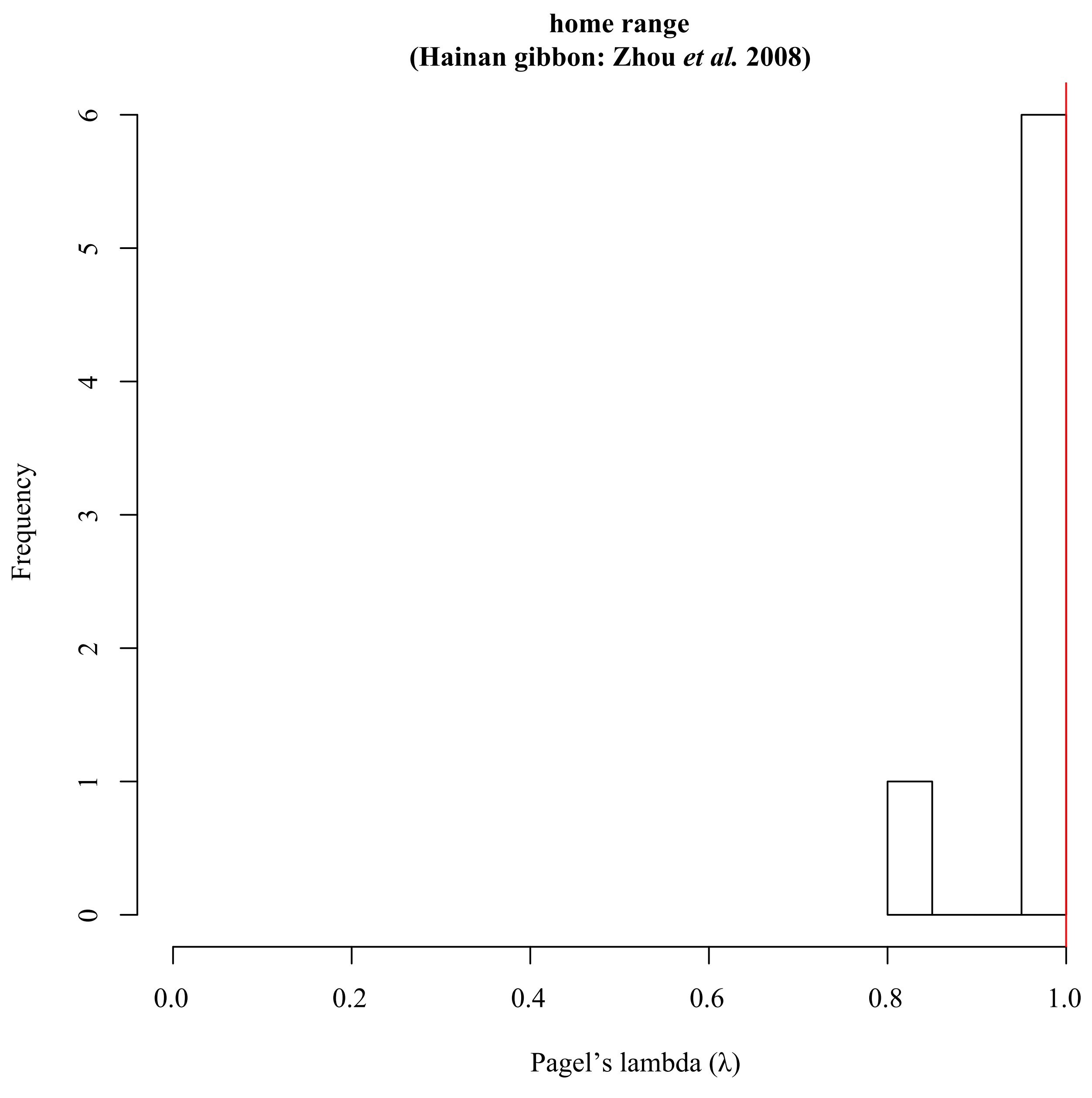
home range
(Hainan gibbon: Zhou et al. 2008)
Frequency
0
1
2
3
4
5
6
0.0
0.2
0.4
0.6
0.8
1.0
Pagel’s lambda (λ)

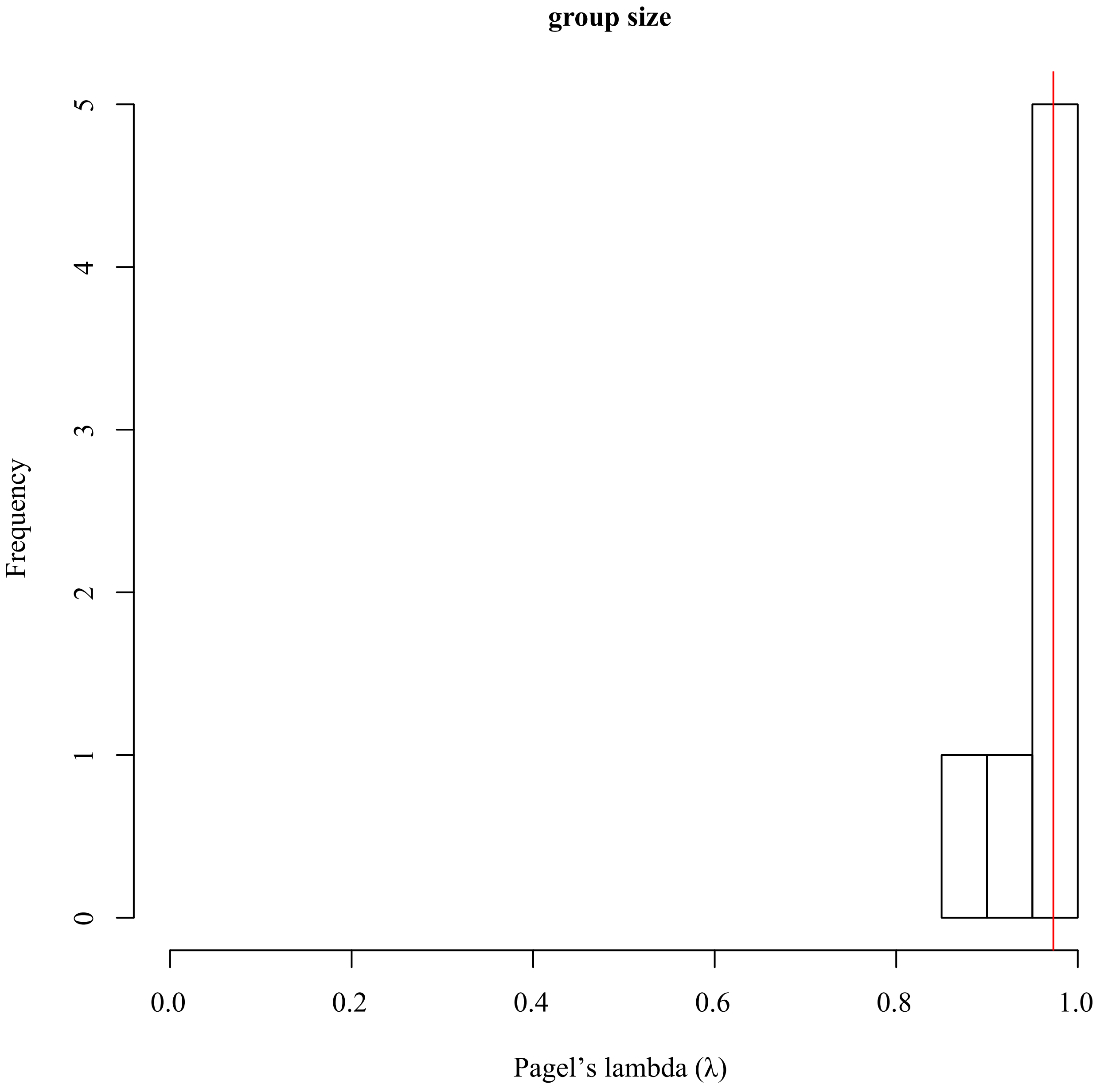
group size
Frequency
0
1
2
3
4
5
0.0
0.2
0.4
0.6
0.8
1.0
Pagel's lambda (λ)

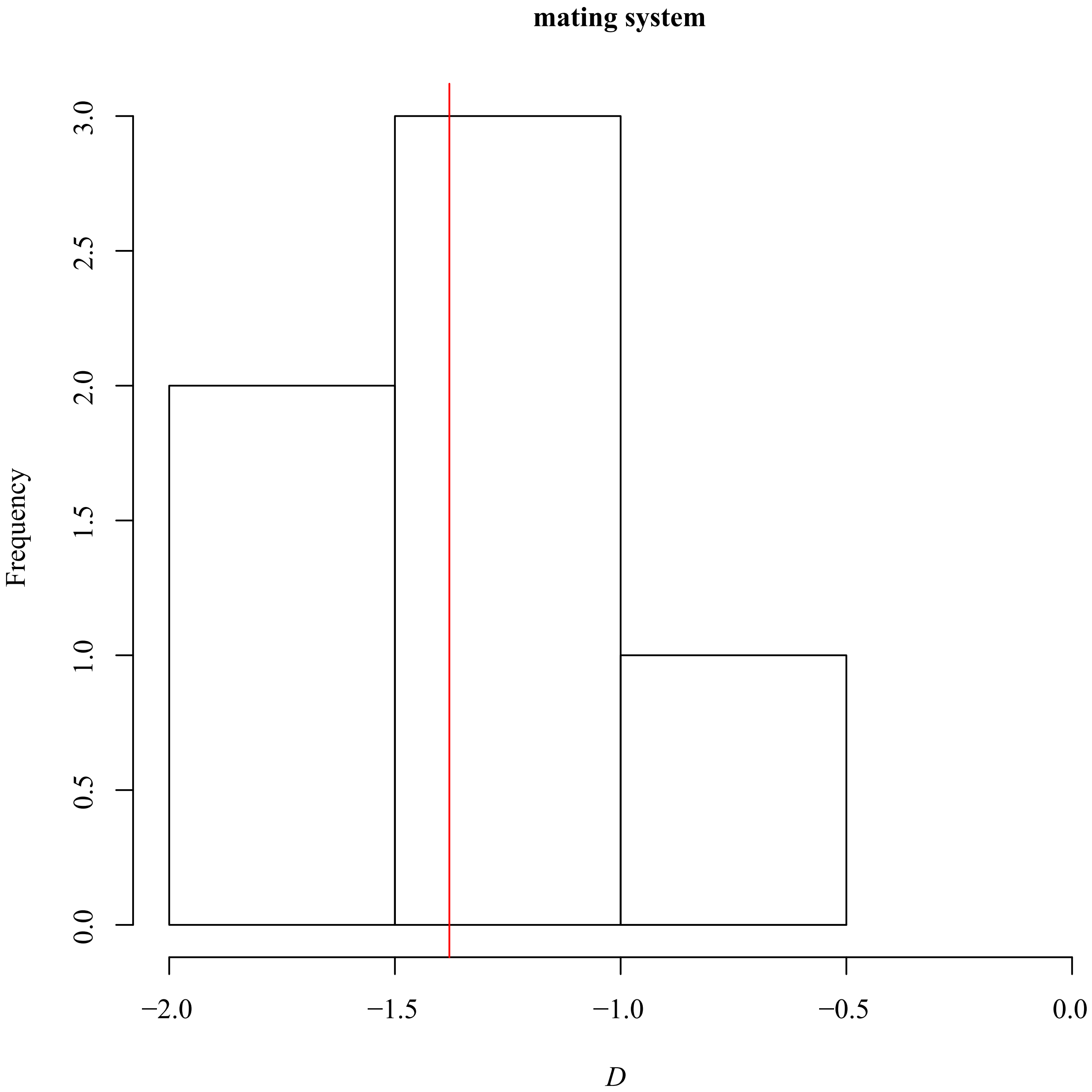
mating system
Frequency
0.0
0.5
1.0
1.5
2.0
2.5
3.0
−2.0
−1.5
−1.0
−0.5
0.0
D
