# Supplementary material for: Identifying environmental versus phylogenetic correlates of behavioural ecology in gibbons: implications for conservation management of the world’s rarest ape
Source: BMC Evol Biol. 2015 Aug 25;15:171. doi: 10.1186/s12862-015-0430-1 (PMC4549120; doi:10.1186/s12862-015-0430-1)
Supplement: Additional file 3: — Global multiple regression linear mixed-effects kinship models incorporating all significant predictors ( P < 0.05) from separate single regression models for: a) home range and b) group size. Significant P-values in bold. (DOCX 19 kb) [file 12862_2015_430_MOESM3_ESM.docx]

# Additional file 3 Global multiple regression linear mixed-effects kinship models incorporating all significant predictors (*P*<0.05) from separate single regression models for a) home range and b) group size. Significant P-values in bold.

**a) Home range global model (residual error: 0.114)**

| Fixed effect parameter estimates | |  |  |  |
| --- | --- | --- | --- | --- |
| **Coefficient** | **Estimate** | **SE** | **z-value** | ***P*-value** |
| (Intercept) | 1.65 | 0.63 | 2.63 | **0.0085** |
| group size | 0.95 | 0.43 | 2.24 | **0.025** |
| group density | -0.11 | 0.05 | -2.24 | **0.025** |
| mating system (1=polygyny) | 0.24 | 0.12 | 1.89 | 0.059 |
| annual mean temperature | -0.01 | 0.01 | -0.81 | 0.420 |
| annual precipitation | -0.12 | 0.17 | -0.74 | 0.460 |

| Random effects |  |
| --- | --- |
| Variance explained by phylogeny (%) | 99.15% |
| Variance explained by within-species variation (%) | 0.85% |
| Total variance explained: | 9.84E-05 |

**b) Group size global model (residual error: 0.043)**

| Fixed effect parameter estimates | |  |  |  |
| --- | --- | --- | --- | --- |
| **Coefficient** | **Estimate** | **SE** | **z-value** | ***P*-value** |
| (Intercept) | 0.64 | 0.25 | 2.51 | **0.012** |
| mating system (1=polygyny) | 0.16 | 0.04 | 4.25 | **0.00002** |
| home range | 0.11 | 0.06 | 2.07 | **0.039** |
| annual precipitation | -0.12 | 0.06 | -2.04 | **0.042** |
| annual mean temperature | 0.01 | 0.00 | 1.4 | 0.160 |
| latitude | 0.00 | 0.00 | -0.16 | 0.870 |

| Random effects |  |
| --- | --- |
| Variance explained by phylogeny (%) | 18.55% |
| Variance explained by within-species variation (%) | 81.45% |
| Total variance explained: | 2.51E-08 |
