# Supplementary material for: Identifying environmental versus phylogenetic correlates of behavioural ecology in gibbons: implications for conservation management of the world’s rarest ape
Source: BMC Evol Biol. 2015 Aug 25;15:171. doi: 10.1186/s12862-015-0430-1 (PMC4549120; doi:10.1186/s12862-015-0430-1)
Supplement: Additional file 4: — Ranking of candidate models (representing all possible combinations of the five predictors in each global model) by AICc, along with relative log-likelihood (RLL), ΔAICc, and model Akaike weights ( w i ) for: a) home range and b) group size. (DOCX 25 kb) [file 12862_2015_430_MOESM4_ESM.docx]

# Additional file 4 Ranking of candidate models (representing all possible combinations of the five predictors in each global model) by AICc, along with relative log-likelihood (RLL), ΔAICc, and model Akaike weights (*w_i_*) for:

# Home range

| **Candidate model** | **RLL** | **AICc** | **ΔAICc** | ***w_i_*** |
| --- | --- | --- | --- | --- |
| 1 | 1.00 | -36.79 | 0.00 | 0.21 |
| 2 | 0.69 | -36.06 | 0.73 | 0.15 |
| 3 | 0.62 | -35.84 | 0.95 | 0.13 |
| 4 | 0.43 | -35.08 | 1.71 | 0.09 |
| 5 | 0.32 | -34.49 | 2.30 | 0.07 |
| 6 | 0.21 | -33.68 | 3.11 | 0.04 |
| 7 | 0.19 | -33.45 | 3.35 | 0.04 |
| 8 | 0.18 | -33.42 | 3.38 | 0.04 |
| 9 | 0.17 | -33.29 | 3.50 | 0.04 |
| 10 | 0.16 | -33.16 | 3.63 | 0.03 |
| 11 | 0.14 | -32.83 | 3.96 | 0.03 |
| 12 | 0.13 | -32.68 | 4.11 | 0.03 |
| 13 | 0.10 | -32.09 | 4.70 | 0.02 |
| 14 | 0.08 | -31.82 | 4.97 | 0.02 |
| 15 | 0.08 | -31.82 | 4.98 | 0.02 |
| 16 | 0.06 | -31.02 | 5.77 | 0.01 |
| 17 | 0.04 | -30.22 | 6.58 | 0.01 |
| 18 | 0.03 | -29.98 | 6.81 | 0.01 |
| 19 | 0.03 | -29.85 | 6.94 | 0.01 |
| 20 | 0.03 | -29.73 | 7.06 | 0.01 |
| 21 | 0.03 | -29.55 | 7.24 | 0.01 |
| 22 | 0.01 | -28.08 | 8.71 | 0.00 |
| 23 | 0.00 | -23.89 | 12.90 | 0.00 |
| 24 | 0.00 | -23.28 | 13.52 | 0.00 |
| 25 | 0.00 | -17.93 | 18.87 | 0.00 |
| 26 | 0.00 | -16.37 | 20.43 | 0.00 |
| 27 | 0.00 | -16.29 | 20.50 | 0.00 |
| 28 | 0.00 | -15.24 | 21.56 | 0.00 |
| 29 | 0.00 | -13.30 | 23.49 | 0.00 |
| 30 | 0.00 | -10.51 | 26.28 | 0.00 |
| 31 | 0.00 | -7.95 | 28.84 | 0.00 |

# Group size

| **Candidate model** | **RLL** | **AICc** | **ΔAICc** | ***w_i_*** |
| --- | --- | --- | --- | --- |
| 1 | 1.00 | -111.89 | 0.00 | 0.32 |
| 2 | 0.81 | -111.46 | 0.43 | 0.26 |
| 3 | 0.43 | -110.18 | 1.70 | 0.14 |
| 4 | 0.37 | -109.90 | 1.99 | 0.12 |
| 5 | 0.12 | -107.72 | 4.16 | 0.04 |
| 6 | 0.07 | -106.63 | 5.26 | 0.02 |
| 7 | 0.05 | -106.04 | 5.85 | 0.02 |
| 8 | 0.05 | -105.73 | 6.16 | 0.01 |
| 9 | 0.04 | -105.28 | 6.61 | 0.01 |
| 10 | 0.04 | -105.22 | 6.67 | 0.01 |
| 11 | 0.03 | -104.74 | 7.15 | 0.01 |
| 12 | 0.03 | -104.74 | 7.15 | 0.01 |
| 13 | 0.03 | -104.66 | 7.23 | 0.01 |
| 14 | 0.02 | -104.26 | 7.63 | 0.01 |
| 15 | 0.02 | -103.74 | 8.15 | 0.01 |
| 16 | 0.01 | -102.74 | 9.15 | 0.00 |
| 17 | 0.00 | -95.90 | 15.99 | 0.00 |
| 18 | 0.00 | -95.64 | 16.25 | 0.00 |
| 19 | 0.00 | -94.09 | 17.80 | 0.00 |
| 20 | 0.00 | -93.95 | 17.94 | 0.00 |
| 21 | 0.00 | -93.91 | 17.98 | 0.00 |
| 22 | 0.00 | -93.90 | 17.99 | 0.00 |
| 23 | 0.00 | -92.11 | 19.78 | 0.00 |
| 24 | 0.00 | -91.95 | 19.94 | 0.00 |
| 25 | 0.00 | -86.42 | 25.47 | 0.00 |
| 26 | 0.00 | -85.90 | 25.99 | 0.00 |
| 27 | 0.00 | -84.61 | 27.28 | 0.00 |
| 28 | 0.00 | -84.43 | 27.45 | 0.00 |
| 29 | 0.00 | -84.15 | 27.74 | 0.00 |
| 30 | 0.00 | -83.60 | 28.28 | 0.00 |
| 31 | 0.00 | -82.53 | 29.36 | 0.00 |
